# ZmmiR1432‐ *ZmCML21* ‐ *ZmPMA2* Module Affects Maize Low Phosphate Tolerance via Regulating Organic Acid Secretion

**DOI:** 10.1111/pbi.70385

**Published:** 2025-10-06

**Authors:** Laming Pei, Yaqing Yang, Zhe Wang, Wencheng Duan, Ning Liu, Zhaohua Ding, Hui Li

**Affiliations:** ^1^ School of Biological Science and Technology University of Jinan Jinan China; ^2^ Shandong Zhongnong Tiantai Seed Co. Ltd Linyi China; ^3^ Maize Research Institute of Shandong Academy of Agricultural Science Jinan China

**Keywords:** low Pi tolerance, maize, organic acid secretion, plasma membrane H^+^‐ATPase, *ZmCML21*, ZmmiR1432

## Abstract

Phosphorus is indispensable in agricultural production. The growing global demand for food necessitates the development of crops with enhanced phosphorus utilisation efficiency. However, the molecular mechanisms coordinating phosphorus utilisation efficiency in plants remain incompletely characterised. MicroRNAs, pivotal regulators of plant developmental and physiological processes, have emerged as key targets for deciphering the regulatory networks underlying low phosphate (Pi) tolerance. Herein, we delineate the regulatory role of ZmmiR1432 in maize and elucidate its mechanistic basis in conferring low Pi tolerance. Suppression of ZmmiR1432 markedly improved tolerance to Pi deficiency via enhanced organic acid exudation, whereas its overexpression had the opposite effect. It is also indicated that ZmmiR1432 regulates low Pi tolerance through direct modulation of its target gene, *ZmCML21*, a calmodulin‐like protein coding gene that also plays a key role in organic acid secretion and Pi‐deficiency response. Metabolomic and transcriptomic analyses revealed that overexpression of *ZmCML21* severely affected organic acid secretion and altered the expression of genes involved in the citrate cycle (TCA cycle). Furthermore, it is demonstrated that ZmCML21 directly interacts with plasma membrane H^+^‐ATPase (ZmPMA2). Overexpression of *ZmPMA2* phenocopied the ZmmiR1432 knockdown plants and *ZmCML21* overexpression plants. Collectively, our findings uncover a ZmmiR1432‐*ZmCML21* regulatory module that governs low Pi tolerance by modulating ZmPMA2 activity, thereby influencing organic acid secretion and ultimately determining Pi use efficiency. These results provide mechanistic insights and actionable genetic targets for improving Pi use efficiency in maize through molecular breeding and genetic engineering.

## 1 Introduction

Phosphorus (P) is one of the major nutrients for plant growth and developmental processes, ultimately determining agricultural productivity in the global agroecological system. While plants predominantly assimilate and utilise phosphorus as inorganic phosphate (Pi), the availability of Pi is severely restricted due to conversion to insoluble mineral complexes or organic compounds (Holford 1997). Consequently, phosphate (Pi) availability is one of the main limiting factors for crop production systems (Péret et al. 2011; Lopez‐Arredondo et al. 2014).

Maize ( *Zea mays* L.), a globally indispensable staple crop, serves as both an important food and feed crop and a critical raw material for various industrial processes. Both the yield and quality of maize are severely affected by various nutrient deficiencies, among which low Pi availability contributes significantly to the reduction of maize yield (Nuss and Tanumihardjo 2010). In response to Pi deficiency, plants have evolved a number of adaptive strategies to cope with inadequate phosphate conditions, including enhancing the acquisition of external phosphorus, reducing the consumption of internal phosphorus, and enhancing the recycling of internal phosphorus (Ticconi and Abel 2004; Lopez‐Arredondo et al. 2014). Among plant low Pi adaptive responses, rhizosphere acidification constitutes a pivotal mechanism for releasing available Pi from soil insoluble mineral complexes. This process is mediated through the coordinated exudation of organic acids concomitant with proton extrusion (Vance et al. 2003; Zhang et al. 2004). The release of organic acids (mainly citric acid) is coupled to proton efflux (Neumann and Römheld 1999; Yan et al. 2002). It was found that there is a close link between the burst of citrate exudation and plasma H^+^‐ATPase catalysed proton efflux (Tomasi et al. 2009; Zhu et al. 2005).

MicroRNAs (miRNAs) act as pivotal regulators of gene expression through post‐transcriptional gene silencing, translational repression, or heterochromatin modification (Llave et al. 2002). Mounting evidence supports the critical roles of miRNAs in plant adaptation to phosphate (Pi) deprivation. Arabidopsis miR399 was the first miRNA demonstrated to be involved in Pi‐deficiency responses; it regulates Pi homeostasis in Arabidopsis, rice, and soybean by suppressing a ubiquitin‐conjugating E2 enzyme, PHOSPHATE2 (PHO2) (Fujii et al. 2005; Chiou et al. 2006; Pant et al. 2008; Hu et al. 2011). Importantly, Pi deficiency upregulates a maize lncRNA1 (PILNCR1) that attenuates the effects of miR399 on low Pi responses in maize by inhibiting ZmmiR399‐guided cleavage of *ZmPHO2* (Wang et al. 2023). In maize, ZmmiR528 was reported to regulate low Pi tolerance by negatively regulating *ZmLac3* (Pei et al. 2024). Furthermore, miR827 was shown to regulate Pi homeostasis in a nitrate‐dependent pattern by targeting the Nitrogen Limitation Adaptation (*NLA*) gene in Arabidopsis (Kant et al. 2011). Diverse miRNAs involved in adaptive responses to Pi deprivation were identified using small RNA sequencing (Hsieh et al. 2009; Pant et al. 2009; Pei et al. 2013; Li et al. 2016).

Previously, we had identified differently expressed miRNAs between low Pi‐tolerant mutant and wild‐type maize genotypes under different Pi conditions by small RNA deep sequencing (Pei et al. 2013). Among the candidate miRNAs, miR1432 showed higher expression in wild‐type maize genotype than in low Pi‐tolerant mutant, and was significantly suppressed by low Pi stress, suggesting a potential involvement of miR1432 in regulating maize low Pi tolerance. Recent studies have shown that OsmiR1432 could target the acyl‐CoA thioesterase gene, and thus affect grain weight in rice by regulating the rate of grain filling (Zhao et al. 2019). In addition, OsmiR1432 was also found to regulate rice drought stress tolerance by targeting the *CALMODULIN‐LIKE2* gene (Luo et al. 2024) and negatively modulate rice abiotic stress tolerance by suppressing Ca^2+^‐ATPase genes (Dai et al. 2024). But its functional roles in maize low Pi tolerance remain hitherto unknown and need more comprehensive evaluation. In this study, we identified a ZmmiR1432‐*ZmCML21*‐*ZmPMA2* regulatory module that modulates rhizosphere acidification through organic acid secretion, ultimately governing Pi acquisition efficiency. This module provides both conceptual advances in plant nutrient sensing mechanisms and actionable targets for precision breeding of low Pi‐tolerant maize.

## 2 Results

### 2.1 Expression of ZmmiR1432 Under Pi Starvation

**FIGURE 1:**
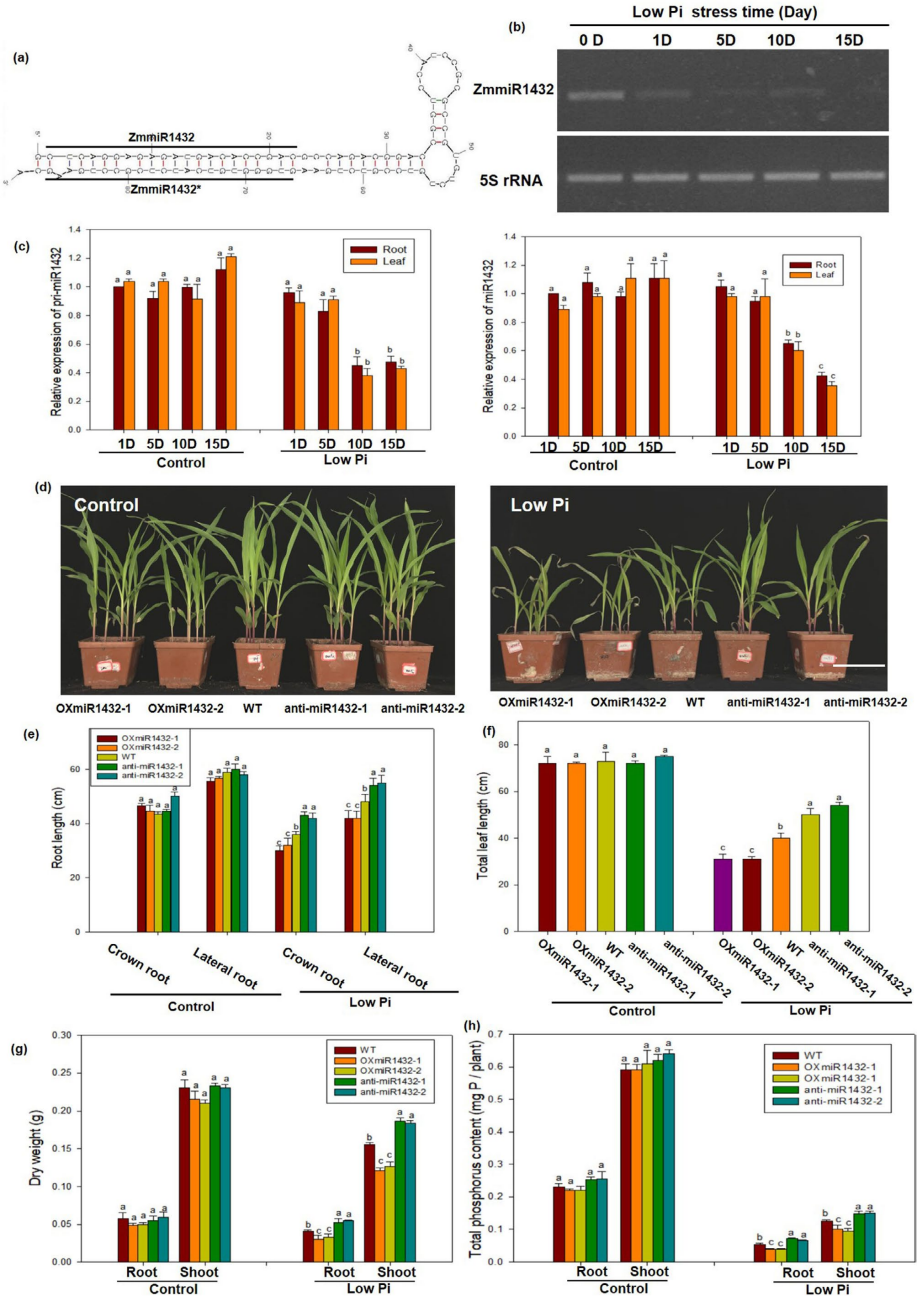
ZmmiR1432 negatively regulated maize low Pi tolerance. (a) The hairpin‐loop structure of pre‐miR1432 in maize plants. The secondary structure of pre‐miR1432 was predicted by Mfold. ZmmiR1432* is a by‐product of the miRNA:miRNA* duplex. (b) Relative expression level of ZmmiR1432 responsive to low Pi stress. 7‐day‐old maize seedlings were transferred to low phosphate (5 μM KH_2_PO_4_) nutrient solution. After different stress times (0, 1, 5, 10, 15 days), root tips of maize plants were collected to analyse gene expression levels by RT‐PCR. The expression levels of ZmmiR1432 was normalised to that of maize 5S rRNA. (c) Expression pattern of ZmmiR1432 and pri‐miR1432 responsive to low Pi stress. Maize seeds were surface sterilised and germinated, then transferred into sufficient phosphate (Control, 1 mM KH_2_PO_4_) or low phosphate (low Pi stress, 5 μM KH_2_PO_4_) nutrient solution for 7 days. Then after different stress times (1, 5, 10, 15 days), root tips and leaves of maize plants were collected to analyse gene expression levels by real‐time PCR. The expression levels of ZmmiR1432 and primiR1432 were normalised to that of maize 5S rRNA and maize *Actin1*, respectively. To analyse low Pi tolerance, maize seeds were sown in low Pi soil (total phosphate concentration is about 0.83 g/kg soil, available phosphate concentration is about 7.13 mg/kg soil), and maize plants watered with sufficient phosphate (1 mM KH_2_PO_4_) nutrient solutions acted as control. After 3 weeks of growth, the photographs were taken. Scale bar indicates 15 cm (d), root length (e), leaf length (f), biomass (g) and Pi content (h) were measured. Different lowercase letters indicate the statistically significant difference in same tissue between maize plants under same Pi conditions at the *p* < 0.05 level using Duncan's multiple‐range test. Values are means ± SD of three biological replicates.

The secondary structure of pre‐miR1432 was predicted by Mfold; the result indicated that pre‐miR1432 could form a hairpin‐loop structure with the mature ZmmiR1432 residing in the stem region (Figure 1a), which is a structure necessary for microRNA maturation. As shown in Figure 1b, the expression of ZmmiR1432 was obviously inhibited by low Pi stress. Moreover, the expression level of ZmmiR1432 in roots was similar to that in leaves and significantly downregulated by Pi deficiency, and the expression of pri‐miR1432 displayed a similar trend to mature ZmmiR1432 (Figure 1c). These results indicated that ZmmiR1432 was significantly downregulated by low Pi stress, which implied its potential involvement in maize low Pi adaptive responses.

### 2.2 ZmmiR1432 Negatively Modulates Low Pi Tolerance in Maize

In order to investigate the role of ZmmiR1432 in regulating low Pi tolerance in maize, transgenic maize plants that over‐expressed ZmmiR1432 (OXmiR1432) or down‐expressed ZmmiR1432 (anti‐miR1432) were generated (Figure S1). After 20 days of Pi starvation, anti‐miR1432 lines displayed better growth status than WT and the OXmiR1432 lines (Figure 1d). As shown in Figure 1e,f, anti‐miR1432 plants exhibited significantly higher root length and leaf length compared to WT and OXmiR1432 lines under low Pi stress, but no significant difference was found in leaf anthocyanin content among all lines (Figure S2). In addition, the biomass and Pi content were significantly increased in anti‐miR1432 lines compared to WT and OXmiR1432 lines under low Pi stress (Figure 1g,h). These results indicated that ZmmiR1432 plays an important regulatory role in maize low Pi tolerance.

### 2.3 Identification of the miR1432 Target Genes

**FIGURE 2:**
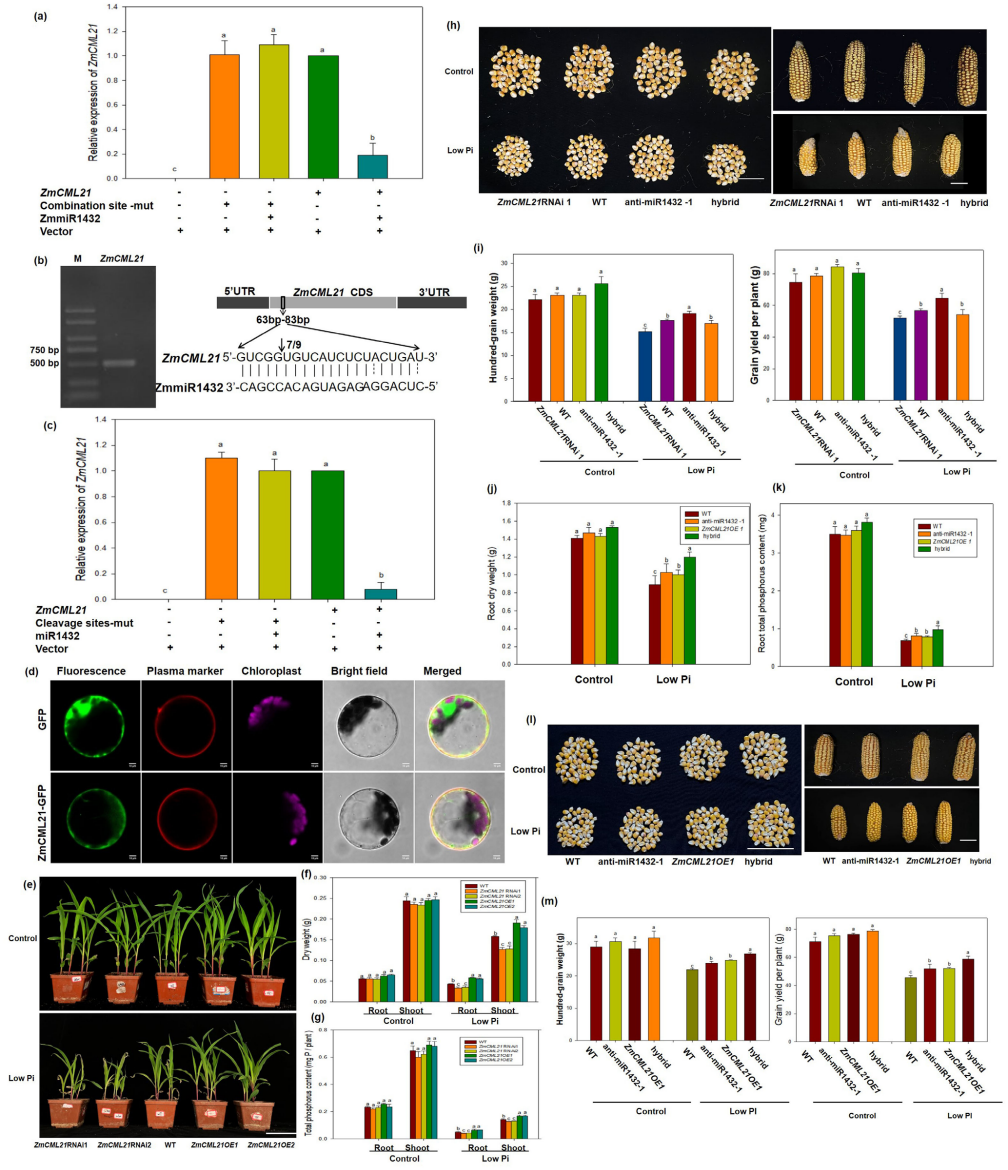
*ZmCML21* plays positive roles in maize low Pi tolerance. (a) Co‐expression assay of ZmmiR1432 and its putative targets (*ZmCML21*) in *Nicotiana benthamiana* leaves. (b) Predicted sites cleavage by ZmmiR1432 in *ZmCML21*. The predicted target sites in *ZmCML21* were determined by performing a 5′ RACE (rapid amplification of cDNA ends). Numbers indicate the frequency of cleavage at each site. (c) Co‐expression assay of ZmmiR1432 and its putative targets (*ZmCML21*) in *Nicotiana benthamiana* leaves. (d) Subcellular localisation of ZmCML21. The subcellular localisation of ZmCML21‐GFP in maize protoplast cells was visualised using confocal laser‐scanning microscopy. Scale bar = 10 μm. GFP, green fluorescent protein. To analyse low Pi tolerance, maize seeds were sown in low Pi soil (total phosphate concentration is about 0.83 g/kg soil, available phosphate concentration is about 7.13 mg/kg soil), and maize plants watered with sufficient phosphate (1 mM KH_2_PO_4_) nutrient solutions acted as control. After three weeks of growth, the photographs were taken, scale bar indicates 15 cm (e), biomass (f) and Pi content (g) were measured. To clarify the genetic relationship between miR1432 and *ZmCML21*, we generated anti‐miR1432:*ZmCML21*RNAi hybrid plants by hybridising anti‐miR1432‐1 and *ZmCML21*RNAi1 transgenic plants. Maize seeds of offspring were sown in low Pi soil (total phosphate concentration is about 0.83 g/kg soil, available phosphate concentration is about 7.13 mg/kg soil) and watered with low phosphate (low Pi, 5 μM KH_2_PO_4_) nutrient solution every 3 days, maize plants watered with sufficient phosphate (1 mM KH_2_PO_4_) nutrient solutions acted as control. At harvest time, photographs of maize grain and ear were taken. Scale bar for grain indicates 5 cm, and for ear indicates 3 cm (h). Hundred‐grain weight and grain yield per plant (i) were measured. Moreover, anti‐miR1432: *ZmCML21OE* hybrid plants were also generated. Maize seeds of offspring were sown in low Pi soil (total phosphate concentration is about 0.83 g/kg soil, available phosphate concentration is about 7.13 mg/kg soil) and watered with low phosphate (low Pi, 5 μM KH_2_PO_4_) nutrient solution every 3 days, maize plants watered with sufficient phosphate (1 mM KH_2_PO_4_) nutrient solutions acted as control. At harvest time, root dry weight (j) and root Pi content (k) were determined. Photographs of maize grain and ear were taken. Scale bar for grain indicates 5 cm. Scale bar for ear indicates 3 cm (l). Hundred‐grain weight and grain yield per plant (m) were measured. Different lowercase letters indicate the statistically significant difference between maize plants under same Pi conditions at the *p* < 0.05 level using Duncan's multiple‐range test. Values are means ± SD of three biological replicates.

The potential targets of ZmmiR1432 were predicted by using the psRNATarget program (http://plantgrn.noble.org/psRNATarget/), based on criteria as described previously (Allen et al. 2005; Meyers et al. 2008; Ma et al. 2018). A calmodulin‐like protein coding gene (*ZmCML21*, Gene ID: 109939414) and a calcium‐transporting ATPase 5 coding gene (Gene ID: 103626686) were suggested to be potential target genes of miR1432 in maize (Table S1). As shown in Figure S3a, the expression pattern of *ZmCML21* is obviously opposite to that of ZmmiR1432 in response to Pi deficiency. However, the expression of the calcium‐transporting ATPase coding gene did not show an obvious negative association with miR1432 in response to Pi deficiency (Figure S3b), indicating that this gene may not be involved in the miR1432‐mediated response to low Pi stress. Moreover, the expression levels of *ZmCML21* were significantly increased in anti‐miR1432 lines and markedly reduced in OXmiR1432 lines relative to those in the WT (Figure S3c). The expression of the calcium‐transporting ATPase coding gene did not exhibit significant changes in anti‐miR1432 lines and OXmiR1432 lines (Figure S3d). In addition, as shown in Figure 2a, the expression of *ZmCML21* was significantly downregulated by ZmmiR1432 in *N. benthamiana* leaves when the synonymous mutations in the miR1432 recognition sites within *ZmCML21* (G TCG GTG TCA TCT CTA CTG AT‐‐‐‐G TCT GTA TCT TCG CTG CTC AT) were introduced, the expression of *ZmCML21* was not affected by ZmmiR1432.

To further validate the interaction between ZmmiR1432 and *ZmCML21*, the ZmmiR1432‐directed cleavage sites were determined in *ZmCML21* by performing RNA ligase‐mediated rapid amplification of cDNA ends (RLM‐RACE). As shown in Figure 2b, cleavage occurred between the 16th and 17th base pairs in *ZmCML21*. To further confirm the cleavage site, the mutation of the cleavage site (GT‐AC) was introduced in *ZmCML21*, and a transient co‐expression experiment in *N. benthamiana* leaves was performed. As shown in Figure 2c, the expression of *ZmCML21* was significantly downregulated by ZmmiR1432 in *N. benthamiana* leaves. When the cleavage sites within *ZmCML21* were mutated, the expression of *ZmCML21* was not affected by ZmmiR1432. Moreover, the potential complementary miRNAs targeting the *ZmCML21* were predicted by using the psRNATarget program, based on criteria as described previously (Allen et al. 2005; Meyers et al. 2008; Ma et al. 2018). The results suggested that only miR1432 was suggested to be the potential miRNA targeting the *ZmCML21* in maize (Table S2).

### 2.4 *ZmCML21* Plays Positive Role in Maize Low Pi Tolerance

Subcellular localisation of ZmCML21 was analysed in maize protoplast cells (Figure 2d). Microscopic images showed that the ZmCML21‐GFP fluorescence was observed in the cytoplasm and plasma membrane (PM), while the fluorescence of green fluorescent protein (GFP) or plasma membrane localisation marker control was observed throughout the cell or in the PM, respectively. The results indicated that ZmCML21 was located in the cytoplasm and PM. To further characterise the role of *ZmCML21* in maize low Pi tolerance, transgenic maize plants overexpressing *ZmCML21* (*ZmCML21OE*) or down‐expressing *ZmCML21* (*ZmCML21*RNAi) were generated (Figure S4). Consistent with the results observed in anti‐miR1432 transgenic maize lines, it was found that *ZmCML21OE* maize lines showed better growth status than WT and *ZmCML21*RNAi lines in low Pi soil (Figure 2e). As shown in Figure 2f,g, the biomass and Pi content were significantly higher in *ZmCML21OE* maize plants than in WT and *ZmCML21*RNAi plants, while *ZmCML21*RNAi plants exhibited a more pronounced sensitivity to low Pi stress compared to the wild type. The biomass and P content were significantly lower in *ZmCML21*RNAi maize plants than in WT.

To clarify the genetic relationship between miR1432 and *ZmCML21*, we generated anti‐miR1432:*ZmCML21*RNAi hybrid plants by the hybridisation of anti‐miR1432‐1 and *ZmCML21*RNAi1 transgenic plants. Maize seeds of offspring were sown in low Pi soil. At harvest time, yield traits were determined. In low Pi soil, anti‐miR1432‐1 plants showed better grain growth than other plants (Figure 2h). As shown in Figure 2i, the hundred‐grain weight and grain yield per plant in anti‐miR1432‐1 plants were significantly higher than those in other maize plants, while the *ZmCML21*RNAi1 plants showed the lowest values in these parameters. The phenotypes of *ZmCML21*RNAi1 plants were restored in anti‐miR1432:*ZmCML21*RNAi hybrid plants to a level similar to WT plants. Moreover, we also generated anti‐miR1432:*ZmCML21OE* hybrid plants. Maize seeds of offspring were sown in low Pi soil. At harvest time, the root dry weight, root phosphorus content, Pi content of rhizosphere soil, and yield traits were determined. As shown in Figure 2j,k, anti‐miR1432:ZmCML21OE hybrid plants exhibited the highest root dry weight and root phosphorus content among all maize lines. No significant difference was found in the available Pi content of rhizosphere soil among all plant lines (Figure S5). Moreover, anti‐miR1432:*ZmCML21OE* hybrid plants showed better grain growth than other plants (Figure 2l). As shown in Figure 2m, the hundred‐grain weight and grain yield per plant in anti‐miR1432:ZmCML21OE hybrid plants were significantly higher than those in other maize plants; the hundred‐grain weight increased by about 9.2%, 10.7%, and 17.6% in anti‐miR1432:*ZmCML21OE* hybrid plants compared to *ZmCML21OE*, anti‐miR1432, and WT, respectively. The grain yield per plant increased by about 16.4%, 14.1%, and 25.2% in anti‐miR1432:*ZmCML21OE* hybrid plants compared to *ZmCML21OE*, anti‐miR1432, and WT, respectively, suggesting the potential value in maize breeding in low Pi soil. The anti‐miR1432 plants disrupt the stability of endogenous miR1432 through antisense RNA interference, thereby relieving its inhibition of *ZmCML21*. *ZmCML21OE* plants drive overexpression of *ZmCML21* through a strong promoter. While anti‐miR1432:ZmCML21OE hybrid plants inherit two regulatory advantages simultaneously, this might be the reason why anti‐miR1432:ZmCML21OE hybrid plants exhibited better grain growth and grain yield than anti‐miR1432 and *ZmCML21OE* under low Pi conditions.

### 2.5 Metabolome and Transcriptome Analysis

**FIGURE 3:**
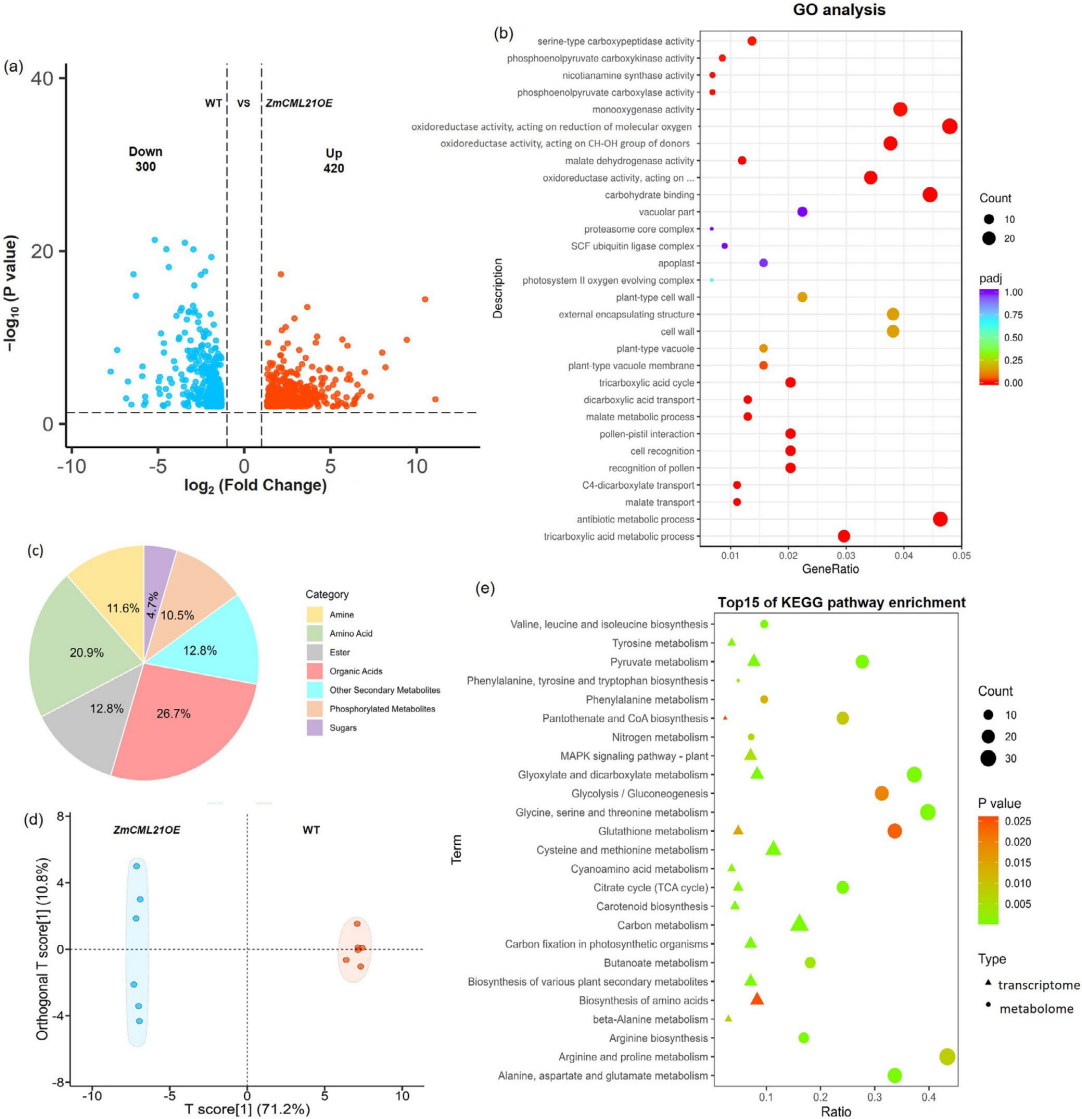
Transcriptome analysis and metabolome analysis between WT and *ZmCML21OE* plants. (a) Volcano plots of the DEGs between WT and *ZmCML21OE* plants revealed by the RNA‐seq analysis. Red dots represent significantly upregulated, and blue dots represent significantly downregulated genes. (b) GO annotations pathway enrichment analysis of DEGs between WT and *ZmCML21OE*. (c) Classification of root exudates metabolites which were differently accumulated between WT and *ZmCML21OE*. (d) Orthogonal Partial Least Squares‐Discriminant Analysis (OPLS‐DA) of root exudates metabolites which were differently accumulated between WT and *ZmCML21OE*. The *x*‐axis represents the predicted component score values, and the *y*‐axis represents orthogonal component score values, respectively. (e) Association analysis of significant enriched KEGG pathway of metabolome and transcriptome. To explore the correlation between DEGs and differently accumulated metabolites between WT and *ZmCML21OE*, association analysis was conducted to display the distribution of two types of data in the KEGG pathway using the ggplot2 R package. Based on the enrichment ratio (Ratio), number of genes and metabolites (Count), and significance level (*p*‐value), the pathways are screened and compared. The top 15 most significantly enriched KEGG pathways are displayed, where the size and colour of nodes in the graph represent the number and significance level of participating pathways, respectively.

The results of transcriptome analysis showed that approximately 700 functionally annotated genes were differentially expressed between *ZmCML21OE1* and WT under Pi deficiency (Table S3). Among these, 420 genes were upregulated and 300 genes were downregulated (Figure 3a). It is worth noting that lots of genes involved in organic acid synthesis and metabolism were significantly differently expressed between *ZmCML21OE1* and WT, such as two malic enzyme coding genes that were expressed at significantly lower levels in *ZmCML21OE1* plants than in WT, and four malate dehydrogenase coding genes and four phosphoenolpyruvate carboxylase coding genes were expressed at significantly higher levels in *ZmCML21OE1* plants than in WT (Table S3). To validate the transcriptome data, we have performed qPCR of eight key differentially expressed genes involved in organic acid synthesis and metabolism, including citrate synthase, malate dehydrogenase, malic enzyme, and phosphoenolpyruvate carboxylase coding genes. Except for the gene encoding malic enzyme 1 and the gene encoding malic enzyme 4, there was a significant upregulation of these genes in *ZmCML21OE* plants and a downregulation in *ZmCML21*RNAi plants compared with WT (Figure S6).

Gene Ontology (GO) enrichment analysis indicated that lots of differentially expressed genes (DEGs) are involved in carboxylic acid metabolism, malate metabolic process, tricarboxylic acid cycle, citrate metabolic process (Table S4; Figure 3b). Correspondingly, the results of KEGG showed that 13 genes were significantly enriched in pyruvate metabolism and 8 genes were significantly enriched in the citrate cycle (TCA cycle) (Table S5). The results of metabolomics analysis showed that there are approximately 90 metabolites which were differently secreted between *ZmCML21OE1* and WT plants (Table S6), mainly including amine (11.6%), amino acid (20.9%), ester (12.8%), organic acids (26.7%), phosphorylated metabolites (10.5%), sugars (4.7%), others (12.8%) (Figure 3c). OPLS‐DA score plots indicate that statistically significant segregation occurred in the different comparison groups (Figure 3d). It is worth noting that most of the differently accumulated organic acids showed significantly higher contents in *ZmCML21OE* root exudates than in WT, such as malic acid, citric acid, oxalic acid, succinic acid, acetic acid, and fumaric acid, which were reported to be crucial for releasing Pi from insoluble complexes and helpful for low Pi adaptation (Zhang et al. 1997; Wang et al. 2007; Zhou et al. 2016). Moreover, KEGG analysis showed that 20 of the metabolites were enriched in the citrate cycle (TCA cycle) pathway, and 23 of the metabolites were enriched in the pyruvate metabolism pathway (Table S7). These results suggested that the enhanced low Pi tolerance observed in *ZmCML21OE* plants might be contributed to by the improved organic acid secretion. Moreover, to explore the correlation between differently expressed genes and differentially accumulated metabolites, association analysis was conducted to display the distribution of two types of data in the KEGG pathway. As shown in Figure 3e, among the top 15 most significantly enriched pathways of the metabolome and transcriptome, the pyruvate metabolism pathway and the TCA cycle pathway overlapped between the two types of data.

### 2.6 Interaction Analysis of ZmCML21 With ZmPMA2

**FIGURE 4:**
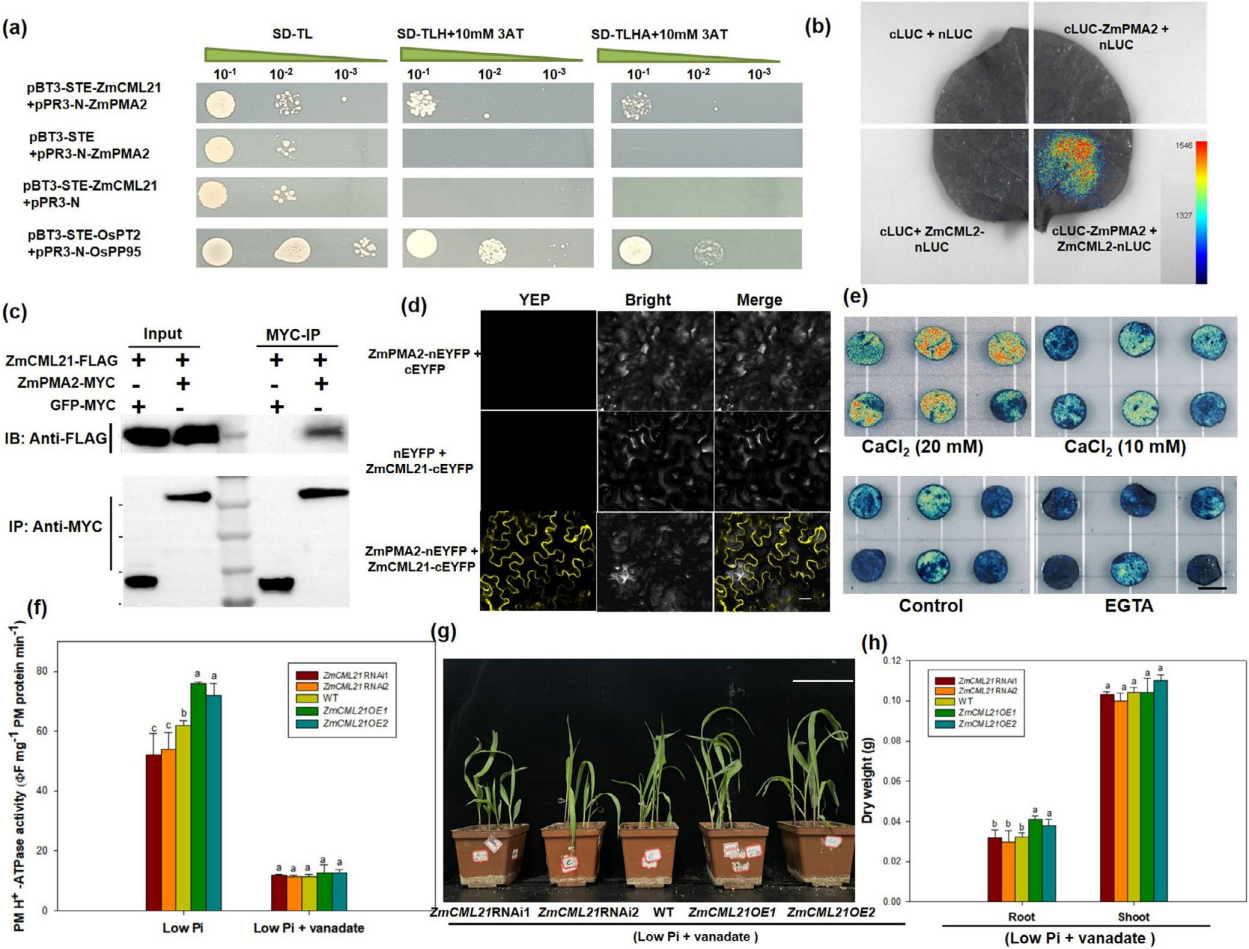
Interaction analysis of ZmCML21 with ZmPMA2. Interaction of ZmCML21 with ZmPMA2 was analysed using the following methods. (a) Y2H. OsPT2, rice phosphate transporter 2; OsPP95, rice purple acid phosphatase 95. pBT3‐STE‐OsPT2 + pPR3‐N‐OsPP95 served as positive controls. 3AT (3‐Amino‐1,2,4‐Triazole), auto‐activation inhibitor. (b) LUC. The luciferase signal was detected after 72 h of co‐infiltration. The combinations of cLUC and nLUC, cLUC‐ZmPMA2 and nLUC, cLUC and ZmCML2‐nLUC were co‐injected as negative controls. (c) Co‐IP. ZmCML21‐FLAG, ZmPMA2‐MYC and empty vector were individually transformed into GV3101, and the transformed strains were infiltrated into *N. benthamiana* leaves, and soluble proteins were extracted. (d) BiFC. ZmPMA2‐nEYFP + cEYFP and nEYFP + ZmCML21‐cEYFP served as negative controls. (e) Analysis of Ca^2+^‐dependent interaction of ZmCML21 with ZmPMA2. Nicotiana benthamiana leaves co‐transformed using the mixed Agrobacterium harbouring 35S::CLUC‐ZmCML21 and 35S::ZmPMA2‐nLUC were placed on 4% agar containing 10 or 20 mM CaCl_2_, 20 mM EGTA or without any supplement as control for 48 h, followed by capturing the luciferase image. To evaluate the effects of PM H^+^‐ATPase on low Pi tolerance in *ZmCML21OE1* plants, maize seeds were sown in low Pi soil (total phosphate concentration is about 0.83 g/kg soil, available phosphate concentration is about 7.13 mg/kg soil), and 500 μM of vanadate was applied to inhibit PM H^+^‐ATPase activity. After 15 days of growth, (f) PM H^+^‐ATPase activity in roots of WT and *ZmCML21* transgenic plants was measured. (g) Photograph of maize plants was taken, scale bar indicates 15 cm. (h) Biomass was measured. Different lowercase letters indicate the statistically significant difference in same tissue between maize plants under same Pi conditions at the *p* < 0.05 level using Duncan's multiple‐range test. Values are means ± SD of three biological replicates.

For further understanding of how *ZmCML21* regulates low Pi tolerance, a yeast two‐hybrid (Y2H) screen was performed, and about 15 functionally annotated proteins were identified to be interacting with ZmCML21 (Table S8). Among them, PM H^+^‐ATPase and ZmPMA2 was selected for further interaction by Y2H analysis. The results showed that ZmPMA2 possibly interacts with ZmCML21 (Figure 4a). The interactions were further confirmed by firefly luciferase complementation imaging (LUC), co‐immunoprecipitation (Co‐IP) assays and bimolecular fluorescence complementation (BiFC). The interactions were observed in the LUC assay (Figure 4b). The interactions between ZmPMA2 and ZmCML21 were further verified using Co‐IP assay (Figure 4c). Co‐expression of ZmCML21‐cEYFP and ZmPMA2‐nEYFP in *Nicotiana benthamiana* leaves resulted in a strong YFP fluorescence in the PM in BiFC assay (Figure 4d). To investigate whether the interaction between ZmCML21 and ZmPMA2 was dependent upon Ca^2+^, a transient LUC assay was performed in the presence of CaCl_2_ or EGTA. The results showed that CaCl_2_‐treated leaves had a stronger interaction signal, while the EGTA‐treated leaves had a weaker signal as compared with the control (Figure 4e), indicating that the interaction between ZmCML21 and ZmPMA2 was dependent upon Ca^2+^. In addition, *ZmCML21OE* lines were found to exhibit significantly higher PM H^+^‐ATPase activity compared with *ZmCML21*RNAi lines and WT, respectively (Figure 4f). Moreover, when PM H^+^‐ATPase activity of all lines was inhibited by exogenously applying vanadate to maize plants, *ZmCML21OE* lines did not show advantages in low Pi tolerance compared to other lines (Figure 4g,h). Given that PM H^+^‐ATPase has been reported to be closely linked to organic acid exudation, which is crucial for low Pi tolerance (Yan et al. 2002; Zhu et al. 2005; Tomasi et al. 2009), these findings suggest that *ZmCML21* may regulate maize low Pi tolerance by influencing organic acid secretion through the regulation of ZmPMA2.

### 2.7 *ZmPMA2* Plays Positive Role in Maize Low Pi Tolerance

**FIGURE 5:**
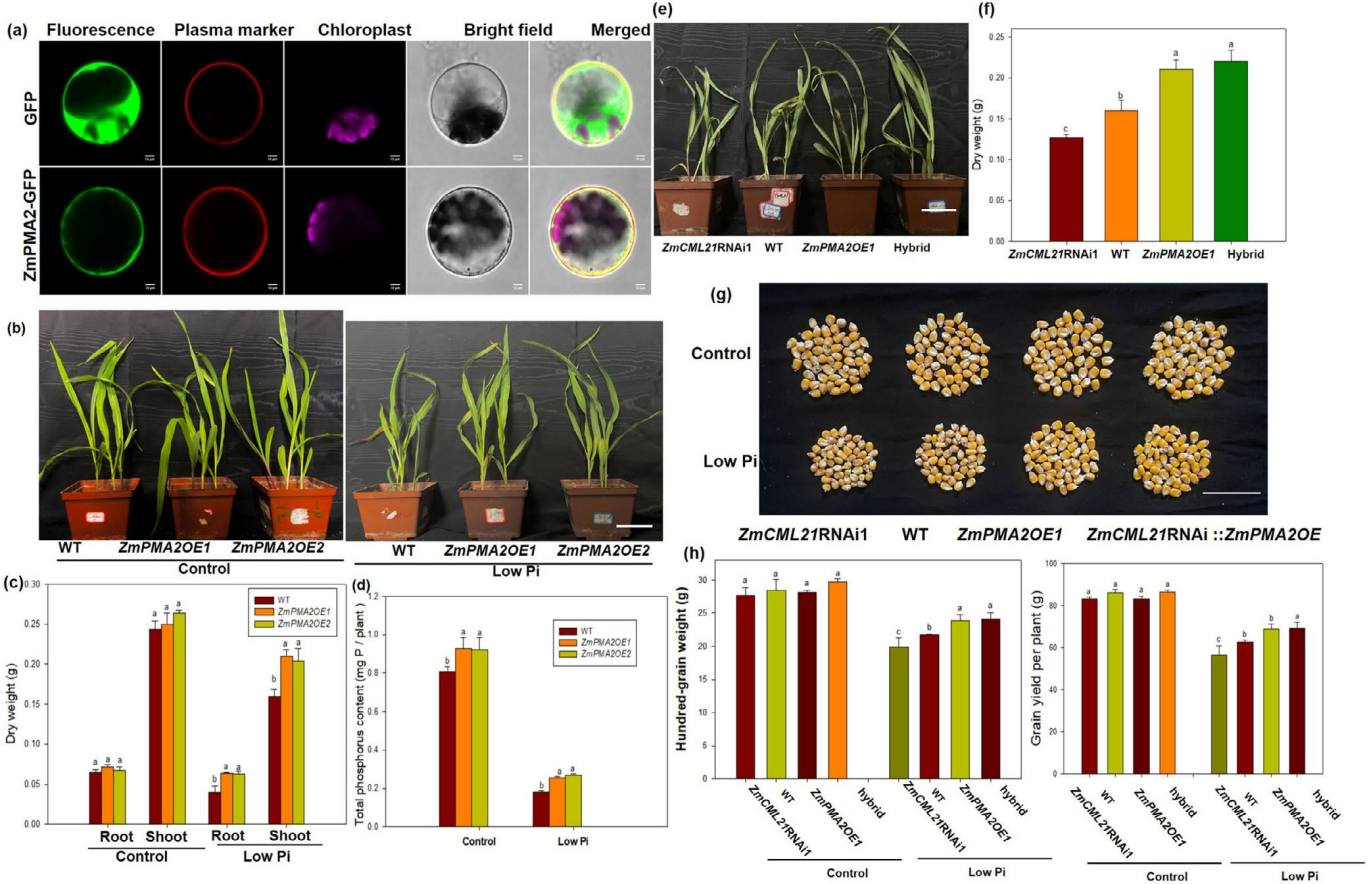
*ZmPMA2* plays positive roles in maize low Pi tolerance. (a) Subcellular localisation of ZmPMA2 under normal conditions (1 mM KH_2_PO_4_). The subcellular localisation of ZmPMA2‐GFP in maize protoplasts cells was visualised using confocal laser‐scanning microscopy. Scale bar = 10 μm. GFP, green fluorescent protein. To analyse the low Pi tolerance of *ZmPMA2OE* transgenic plants, maize seeds were sown in low Pi soil for 3 weeks. And maize plants watered with sufficient phosphate (1 mM KH_2_PO_4_) nutrient solutions acted as control. (b) Phenotypes of *ZmPMA2OE* maize plants and WT. Scale bar indicates 10 cm. (c) Biomass and (d) Pi content. Genetic complementation analysis was conducted through hybridisation of *ZmCML21*RNAi1 and *ZmPMA2OE1* plants. Then maize seeds of offspring were sown in low Pi soil (total phosphate concentration is about 0.83 g/kg soil, available phosphate concentration is about 7.13 mg/kg soil) for 20 days. Then photographs of maize plants were taken, scale bar indicates 10 cm (e) and biomass were measured (f). For yield statistics, maize seeds of offspring were sown in low Pi soil watered with low phosphate (low Pi, 5 μM KH_2_PO_4_) nutrient solution. Maize plants watered with sufficient phosphate (1 mM KH_2_PO_4_) nutrient solutions acted as control. At harvest time, photographs of maize grain were taken, scale bar indicates 5 cm (g). Hundred‐grain weight and grain yield per plant were measured (h). Different lowercase letters indicate the statistically significant difference between maize plants under same Pi conditions at the *p* < 0.05 level using Duncan's multiple‐range test. Values are means ± SD of three biological replicates.

To further characterise the role of *ZmPMA2* in maize low Pi tolerance. Transgenic maize plants overexpressing *ZmPMA2* (*ZmPMA2OE*) were generated (Figure S7a–c). *ZmPMA2OE* lines plants exhibited significantly higher PM H^+^‐ATPase activity than WT under control or low Pi stress (Figure S7d). Subcellular localisation of ZmPMA2 was analysed in maize protoplast cells. Microscopic images showed that the ZmPMA2‐GFP fluorescence was observed in the PM, while the fluorescence of GFP or PM localisation marker control was observed throughout the cell or in the PM, respectively (Figure 5a). Consistent with the enhanced low Pi tolerance observed in *ZmCML21OE* lines, *ZmPMA2OE* lines also showed obviously enhanced low Pi tolerance compared with WT plants (Figure 5b–d). Moreover, genetic complementation analysis was conducted through hybridisation of *ZmCML21*RNAi1 and *ZmPMA2OE1* plants in order to validate the interaction between these two genes. Subsequently, the low Pi tolerance of the offspring was analysed. As shown in Figure 5e,f, *ZmCML21*RNAi1 plants were most sensitive to low Pi stress. However, the phenotype of *ZmCML21*RNAi was obviously restored in *ZmCML21*RNAi:*ZmPMA2OE* hybrid plants, which were similar to *ZmPMA2OE* plants. Moreover, for yield statistics, maize seeds of offspring were sown in low Pi soil until harvest. The *ZmCML21*RNAi plants exhibited more inhibited grain growth than other plants, and the hundred‐grain weight and grain yield per plant were significantly lower in *ZmCML21*RNAi plants than in other plants; the phenotypes of *ZmCML21*RNAi under low Pi stress were obviously restored by hybridising with *ZmPMA2OE* plants (Figure 5g,h).

**FIGURE 6:**
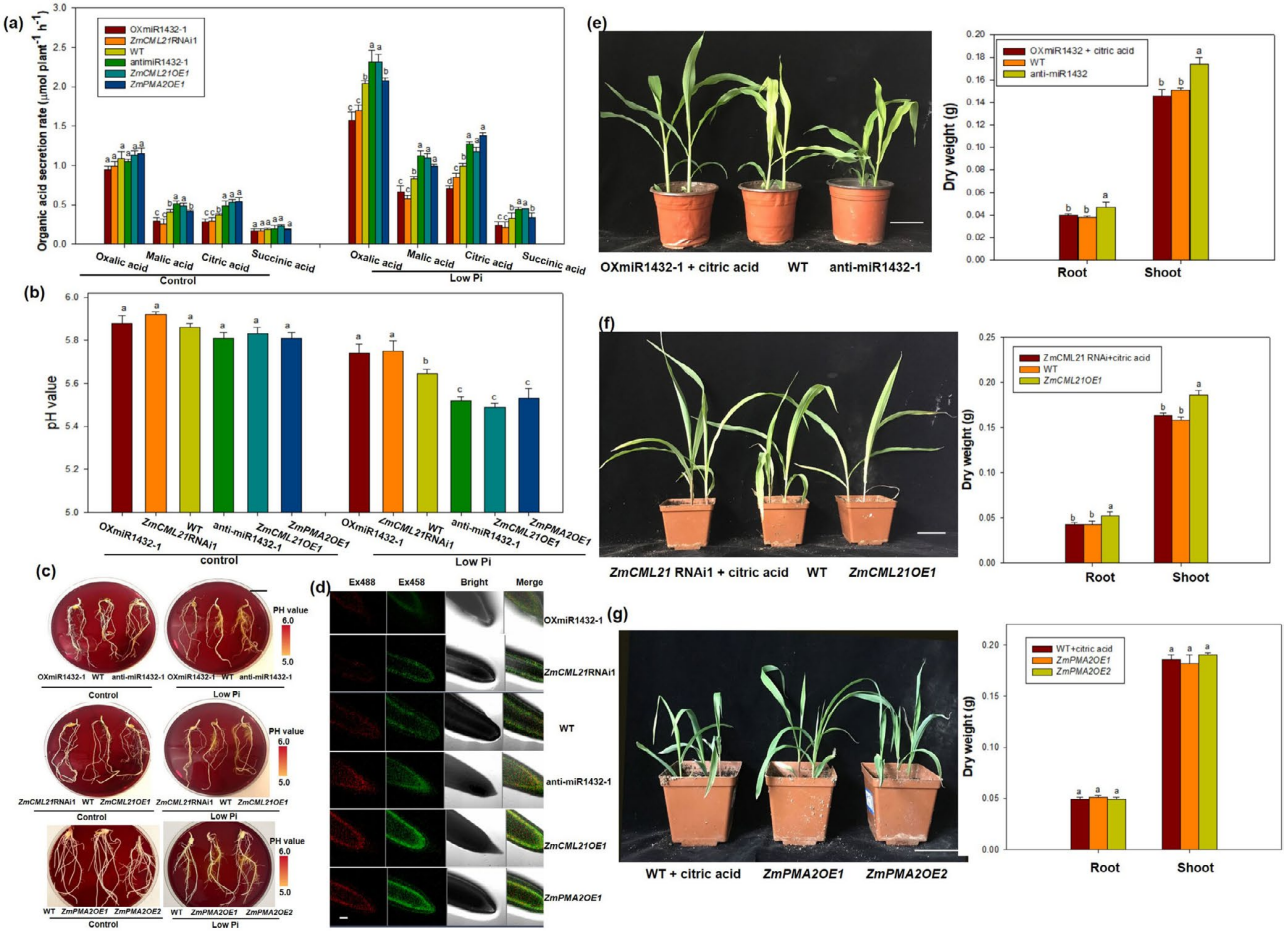
Effects of ZmmiR1432, *ZmCML21* and *ZmPMA2* on root organic acid secretion. Maize seeds were surface sterilised and germinated for 3 days, and then cultured in low phosphate (5 μM KH_2_PO_4_) nutrient solution for 20 days. Then maize root secretion was collected to analysed the contents of organic acid using high‐performance liquid chromatography (HPLC) (a). The pH value of maize culture nutrient solution was measured using portable pH metre (b). Visualisation of rhizosphere acidification was conducted through bromocresol purple pH indicator, scale bar indicated 3 cm (c). Root tip pH fluorescence was detected, scale bar indicates 200 μm (d). To analyse the effects of organic acid on low phosphate tolerance of OXmiR1432, *ZmCML21* RNAi maize plants. Maize seeds were sown in low Pi soil, OXmiR1432 maize plants and *ZmCML21* RNAi maize plants were irrigated with 300 mL of 1 mM citric acid every three days, and others irrigated with water. Two weeks later, the photographs were taken and biomass were determined. (e) Phenotypes and biomass of ZmmiR1432 transgenic maize plants and WT. Scale bar indicates 10 cm. (f) Phenotypes and biomass of *ZmCML21* transgenic maize plants and WT. Scale bar indicates 10 cm. (g) Phenotypes and biomass of *ZmPAM2* transgenic maize plants and WT. Scale bar indicates 10 cm. Different lowercase letters indicate the statistically significant difference in same tissue between maize plants under same Pi conditions at the *p* < 0.05 level using Duncan's multiple‐range test. Values are means ± SD of three biological replicates.

Given the closely associated relationship between PM H^+^‐ATPase and organic acid exudation, which is crucial for low Pi tolerance (Neumann and Römheld 1999; Yan et al. 2002; Zhu et al. 2005; Tomasi et al. 2009), and the interaction regulation relationship between ZmmiR1432, *ZmCML21*, and *ZmPMA2*, we analysed the excretion of organic acid in ZmmiR1432, *ZmCML21*, and *ZmPMA2* transgenic plants. As expected, the secretion rate of oxalic acid, malic acid, citric acid, and succinic acid was significantly higher in anti‐miR1432 plants compared to WT plants and OXmiR1432 plants under Pi deficiency; a similar pattern was observed in *ZmCML21OE* plants. The *ZmPMA2OE* plants showed significantly improved malic acid and citric acid excretion compared to WT (Figure 6a). Correspondingly, the pH value was significantly decreased in anti‐miR1432, *ZmCML21OE*, and *ZmPMA2OE* plants compared to WT (Figure 6b). Conceivably, the rhizosphere of anti‐miR1432, *ZmCML21OE*, and *ZmPMA2OE* plants was noticeably more acidified compared with WT (Figure 6c), and root tips were also markedly more acidified in anti‐miR1432, *ZmCML21OE*, and *ZmPMA2OE* plants compared with WT (Figure 6d). Moreover, exogenous application of organic acid restored low phosphate tolerance of OXmiR1432 and *ZmCML21* RNAi maize plants (Figure 6e,f), and exogenous application of organic acids enhanced the low Pi resistance of WT to the level of *ZmPMA2OE* plants (Figure 6g). These results suggested that ZmmiR1432, *ZmCML21*, and *ZmPMA2* have obvious effects on rhizosphere acidification caused by organic acid secretion, which might explain the regulated low Pi tolerance observed in their transgenic plants.

**FIGURE 7:**
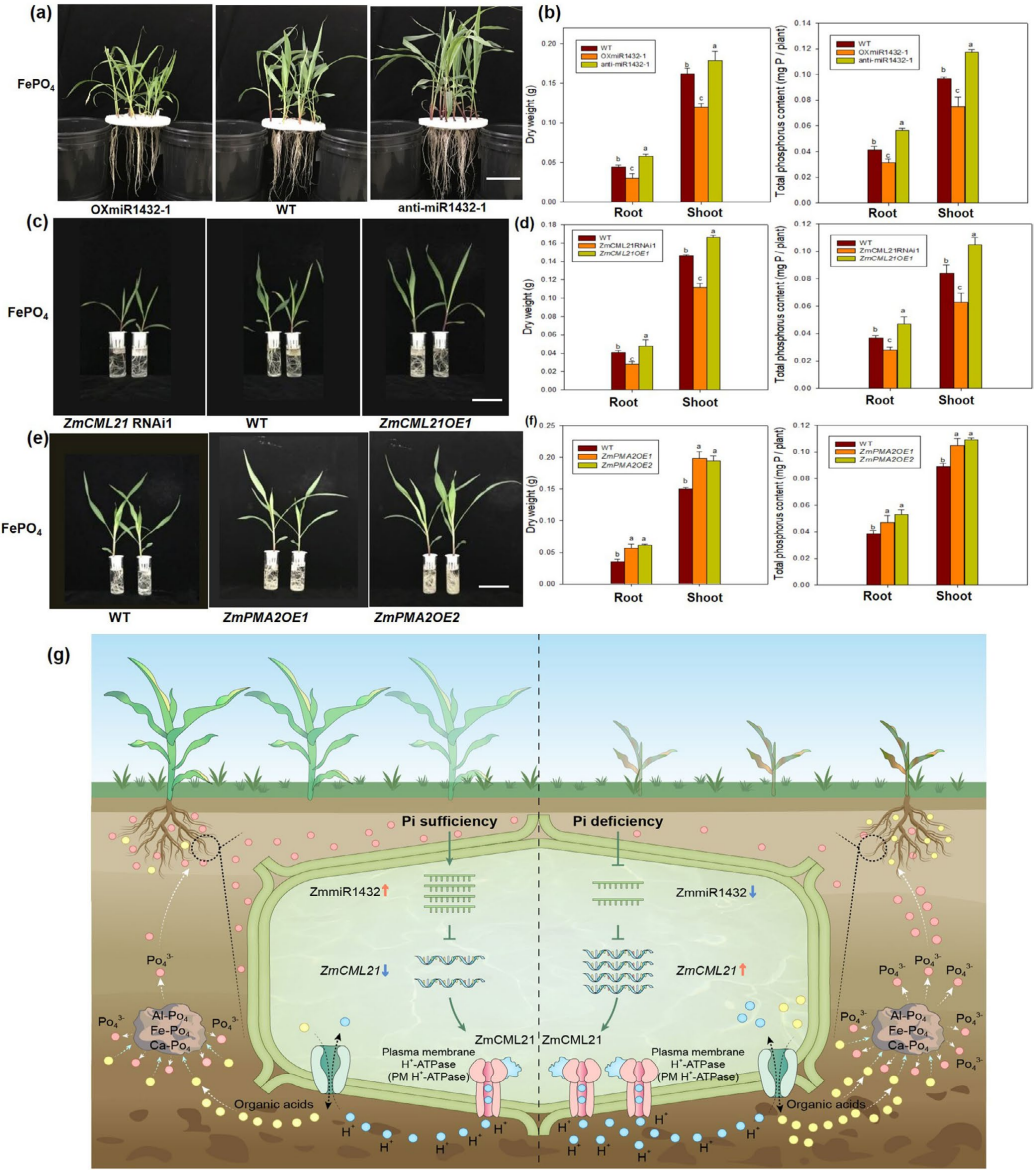
The insoluble phosphate utilisation capacity of ZmmiR1432, *ZmCML21* and *ZmPMA2* transgenic maize plants. Maize seeds were surface sterilised and germinated for 3 days, and then transferred into non‐Pi nutrient solution added FePO_4_ (1 g/L) for 15 days. Maize plants cultured in normal phosphate (Control, 1 mM KH_2_PO_4_) nutrient solutions acted as control. (a) Phenotypes of ZmmiR1432 transgenic maize plants and WT. Scale bar indicates 10 cm. (b) Biomass and Pi content. (c) Phenotypes of *ZmCML21* transgenic maize plants and WT. Scale bar indicates 10 cm. (d) Biomass and Pi content. (e) Phenotypes of *ZmPAM2* transgenic maize plants and WT. Scale bar indicates 10 cm. (f) Biomass and Pi content. Different lowercase letters indicate the statistically significant difference in same tissue between maize plants under same Pi conditions at the *p* < 0.05 level using Duncan's multiple‐range test. Values are means ± SD of three biological replicates. (g) A proposed model for the role of ZmmiR1432‐*ZmCML21* regulatory module in low Pi tolerance in maize. Under Pi sufficient conditions, the expression of ZmmiR1432 is maintained at higher levels, thereby suppressing the target gene, *ZmCML21*, thus the activity of PM H^+^‐ATPase and organic acid secretion is maintained at appropriate levels. Once plants were challenged with low Pi stress, ZmmiR1432 is markedly reduced, thereby alleviating the suppression of *ZmCML21*, which regulates the downstream PM H^+^‐ATPase to promote organic acids secretion, which are crucial for releasing Pi from insoluble complexes for plant assimilation. Arrows indicate positive regulation and blunt‐ended bars indicate negative regulation.

It is known that the enhanced rhizosphere acidification caused by organic acids under low Pi stress is helpful for releasing Pi from insoluble complexes, and then be absorbed and used by plants. Therefore, we tested the insoluble phosphate utilisation capacity of ZmmiR1432, *ZmCML21*, and *ZmPMA2* transgenic maize. Maize plants were cultured in non‐Pi nutrient solution added with FePO4 (0.5 g/L) for 20 days; it was found that the growth of anti‐miR1432 plants was better than that of WT and OXmiR1432 plants (Figure 7a), The biomass and phosphorus content were significantly higher in anti‐miR1432 lines than in WT and OXmiR1432 lines (Figure 7b). The similar trends were observed in *ZmCML21OE* plants and *ZmPMA2OE* plants (Figure 7c–f). These data further confirmed that the ZmmiR1432‐*ZmCML21* module regulates organic acid secretion and ultimately low Pi tolerance by regulating PM H^+^‐ATPases under Pi deficiency.

## 3 Discussion

MicroRNAs are important non‐coding RNAs that are involved in many aspects of plant development and responses to biotic and abiotic stresses (Wang et al. 2009; Song et al. 2019; Zhu et al. 2019; Visentin et al. 2020; Ma et al. 2021). Previous studies have shown that miR1432 could control the rice grain filling rate by targeting acyl‐CoA thioesterase (Zhao et al. 2019). It also could participate in pathogen defence and contribute to rice blast disease and bacterial blight resistance (Jia et al. 2021). Additionally, miR1432 has been reported to regulate rice drought stress tolerance by targeting the *CALMODULIN‐LIKE2* gene and negatively modulate rice abiotic stress tolerance by suppressing Ca^2+^ ATPase gene (Dai et al. 2024; Luo et al. 2024). Despite significant progress in understanding miR1432, the function of the miR1432‐target module in the response of maize to low Pi stress remains poorly understood. We herein reported and verified the functions of the ZmmiR1432‐*ZmCML21*‐*ZmPAM2* module in maize low Pi tolerance.

We found that ZmmiR1432 plays a negative role in maize tolerance to Pi deficiency (Figure 1). Further, we identified the calmodulin‐like gene *ZmCML21* as a direct target of miR1432 (Figures 2a–c and S3). Previous studies have demonstrated that CMLs play important roles in regulating plant growth and development, as well as responses to abiotic and biotic stresses (Reddy et al. 2011; Bender et al. 2013; Yin et al. 2015; Zeng et al. 2015). In the present study, we found that *ZmCML21* plays positive roles in maize low Pi tolerance (Figure 2e–g), further demonstrating the critical role of the miR1432‐*ZmCML21* module in regulating low Pi resistance. CMLs interact with downstream target proteins, thereby regulating their activities and transducing Ca^2+^ signals (Zeng et al. 2015). It has recently been reported that CML regulates plant abiotic stress tolerance by interacting with its downstream target proteins. For instance, CML10 interacts with cytosolic enzymes GSTU8 and FBA6 to regulate cold tolerance in Medicago sativa (Yu et al. 2022). CML15 interacts with BTB and TAZ domain proteins to modulate iron homeostasis in apple (Liu et al. 2024). CML24 interacts with CAMTA2 and WRKY46 to regulate Al resistance in *Arabidopsis thaliana* (Zhu et al. 2022); CML37 interacts with proteasome maturation factor SlUMP1 to influence chilling tolerance in tomato fruit (Tang et al. 2021). In this study, PM H^+^‐ATPase, ZmPMA2, was documented to interact with ZmCML21 and the interaction is dependent upon Ca^2 +^ (Figure 4). Moreover, *ZmPMA2OE* lines also showed obviously enhanced low Pi tolerance compared with WT plants (Figure 5b–d). In addition, genetic complementation analysis showed that the phenotype of *ZmCML21*RNAi was obviously restored in *ZmCML21*RNAi:*ZmPMA2OE* hybrid plants (Figure 5e–h). These results further suggested that *ZmCML21* plays key roles in maize low Pi tolerance by regulating ZmPMA2.

PM H^+^‐ATPases play a key role in Pi‐deficiency‐induced citrate exudation from plant roots (Lino et al. 1998; Morsomme and Boutry 2000). The release of organic acids, mainly citrate, is reported to be coupled to proton efflux (Neumann and Römheld 1999; Yan et al. 2002; Zhu et al. 2005). It was also found that there is a close link between citrate exudation and plasma H^+^‐ATPase catalysed proton efflux (Zhu et al. 2005; Tomasi et al. 2009). It is now clear that organic acids, such as citrate, malate, and oxalate, exuded from roots help Pi uptake by converting insoluble Pi compounds to a soluble form that is more readily absorbed by plants (López‐Bucio et al. 2000; Yan et al. 2002; Ligaba, Shen, et al. 2004; Ligaba, Yamaguchi, et al. 2004). Here, we demonstrated that the secretion rate of oxalic acid, malic acid, citric acid, and succinic acid was significantly higher in anti‐miR1432 plants compared to WT plants and OXmiR1432 plants under Pi deficiency; a similar pattern was observed in *ZmCML21OE* plants. *ZmPMA2OE* plants showed significantly improved malic acid and citric acid excretion compared to WT (Figure 6a). Correspondingly, the pH value was significantly decreased in anti‐miR1432, *ZmCML21OE*, and *ZmPMA2OE* plants compared to WT (Figure 6b), thus leading to increased insoluble phosphate utilisation capacity in these lines (Figure 6e–g). These results suggest that ZmmiR1432, *ZmCML21*, and *ZmPMA2* have obvious effects on rhizosphere acidification caused by organic acid secretion, which might explain the regulated low Pi tolerance observed in their transgenic plants. In addition, the results of metabolomics analysis showed that a number of organic acids exhibited significantly different secretion between *ZmCML21OE1* and WT plants (Table S6), further demonstrating the critical role of the miR1432‐*ZmCML21‐ZmPMA2* module in regulating low Pi tolerance by affecting organic acid secretion.

In summary, our work suggests a potential signalling pathway mediated by the ZmmiR1432‐*ZmCML21* module that is important in maize low Pi tolerance. Under Pi sufficient conditions, the expression of ZmmiR1432 is maintained at higher levels, thereby suppressing the target gene, *ZmCML21*; thus, the activity of PM H^+^‐ATPase and organic acid secretion is maintained at basal levels. Once plants were challenged with low Pi stress, ZmmiR1432 is markedly reduced, thereby alleviating the suppression of *ZmCML21*, which regulates the downstream PM H^+^‐ATPase to promote organic acids secretion, which are crucial for releasing Pi from insoluble complexes for plant assimilation (Figure 7d).

Overall, our findings in this study demonstrate that ZmmiR1432 is a novel regulator of organic acid secretion and ultimately low Pi tolerance by regulating its target gene *ZmCML21* to control PM H^+^‐ATPases under Pi deficiency. It is envisaged that the genetic manipulation of ZmmiR1432 and/or *ZmCML21* expression may provide an effective approach for improving low Pi tolerance in maize.

## 4 Experimental Procedures

### 4.1 Plant Material

Maize materials used in this study were the elite inbred line Qi 319 (wild type, WT) and homozygous transgenic lines in this genetic background, including ZmmiR1432 overexpression lines, ZmmiR1432‐antisense lines, *ZmCML21* overexpression lines, *ZmCML21* RNAi lines, and *ZmPMA2* overexpression lines.

### 4.2 Generation of Transgenic Maize Lines

Total RNA was extracted from roots using TRIzol reagent (TaKaRa) and used as a template for cDNA synthesis using the RT reagent kit (TaKaRa) according to the manufacturer's protocol. The sequence of maize miR1432 precursor (pre‐miR1432, Gene ID: 103318314) from the NCBI (http://www.ncbi.nlm.nih.gov/) was amplified by PCR with gene‐specific primers. The secondary structure of pre‐miR1432 was predicted by Mfold (http://unafold.rna.albany.edu/?q=mfold/RNA‐Folding‐Form).

For miR1432 sense lines, pre‐miR1432 with *Kpn*I and *BamH*I restriction enzyme cutting sites at 5′ and 3′ ends was obtained by PCR using miR1432 sense adapter primers; the PCR product was inserted downstream of maize constitutive ubiquitin promoter PUbi in binary plant vector pCAMBIA3300‐PUbi::MCS‐Tnos‐P35S::*bar* (pCUB). For miR1432 antisense lines, pre‐miR1432 with *BamH*I and *Kpn*I restriction enzyme cutting sites at 5′ and 3′ ends was obtained by PCR using miR1432 antisense adapter primers; the PCR product was also inserted downstream of maize ubiquitin promoter Pubi in plant vector pCUB.

According to the mRNA sequence of a calcium binding protein CML21 coding gene, *ZmCML21* (Gene ID: 109939414) and PM ATPase coding gene, *ZmPMA2* (Gene ID: 103632998), the complete open reading frame (ORF) was produced by PCR using the maize root cDNA as template; the PCR products were inserted into a pGEM T‐easy vector and sequenced.

For *ZmCML21* overexpression lines, *ZmCML21* ORF with *Kpn*I and *BamH*I restriction enzyme cutting sites at 5′ and 3′ ends was inserted downstream of Pubi in plant vector pCAMBIA3300‐PUbi::MCS‐Tnos‐P35S::*bar* (pCUB). For *ZmCML21* RNAi lines, a 300 bp (1–300 bp) fragment of *ZmCML21* encoding sequence was amplified using primers specific for *ZmCML21* to generate the hairpin structures. The PCR fragment was inserted into pTCK303 vector with opposite orientation on both sides of the rice intron. Sense orientation with *BamH*I and *Kpn*I restriction enzyme cutting sites at the 5′ and 3′ ends of the *ZmCML21* fragment, antisense orientation with *Sac*I and *Spe*I restriction enzyme cutting sites at the 5′ and 3′ ends of the *ZmCML21* fragment. The hairpin structure was inserted into the pCUB vector to construct the RNAi plant expression vector pCAMBIA3300‐pUbi::*zmcml21*‐Tnos‐P35S::*bar*.

For *ZmPMA2* overexpression lines, *ZmPMA2* ORF with *EcoR*I and *BamH*I restriction enzyme cutting sites at 5′ and 3′ ends was inserted downstream of Pubi in plant vector pCAMBIA3300‐PUbi::MCS‐Tnos‐P35S::*bar* (pCUB).

The resultant plant vectors were then transformed into *Agrobacterium tumefaciens* strain GV3101 for maize genetic transformation. *Agrobacterium‐*mediated maize transformation and homozygous transgenic lines were obtained as described by Li et al. (2008).

### 4.3 Gene Expression Pattern Analysis

Maize seeds were surface sterilised with 70% (v/v) ethanol and 0.1% (w/v) HgCl_2_, rinsed with sterile water, and germinated at 28°C in darkness for 3 days, then transferred into low phosphate (5 μM KH_2_PO_4_) nutrient solution for 7 days. Then, after different stress times, root tips (1 cm) and leaves (the second leaf from the top) were collected to analyse the expression pattern of target genes. Plants cultured in sufficient phosphate nutrient solutions (1 mM KH_2_PO_4_) acted as controls.

Extraction and expression analysis of small RNAs was performed as described by Pei et al. (2024). The forward primers for miR1432 and 5S rRNA are shown in Table S9. The reverse primers were provided in the miRcute Plus miRNA qPCR Detection Kit (TIANGEN, China).

To analyse the relative expression levels of pri‐miR1432, Total RNA was extracted using TRIzol reagent (TaKaRa) and treated with RNase‐free DNase. The total RNA was used as a template for cDNA synthesis using the oligo dT primer method of the RT reagent kit (TaKaRa). Real‐time PCR was performed on a LightCycler 480 (Roche) with the SYBR Green RT‐PCR Kit (TaKaRa). Gene expression levels of pri‐miR1432 were normalised with the reference gene *Actin1* and evaluated using the 2^−△△Ct^ method (Livak and Schmittgen 2001). The specific primers for pri‐miR1432 are shown in Table S9.

### 4.4 Identification of miR1432 Target Genes

The potential miRNA targets were first predicted using the psRNATarget program (https://www.zhaolab.org/psRNATarget/). The mature miR1432 sequence was subjected to scanning against maize cDNA databases. The procedures and criteria were followed as described previously (Allen et al. 2005; Meyers et al. 2008). *ZmCML21* and maize pre‐miR1432 were constructed in the pBI221 (pCPB) vector, respectively. The resultant plant vectors and the empty vector (EV) were individually transformed into *Agrobacterium tumefaciens* strain GV3101. *ZmCML21* was co‐expressed with pre‐miR1432 or the EV in the leaves of *Nicotiana benthamiana* by *Agrobacterium‐*mediated transient expression as described previously (Li et al. 2008).

### 4.5 Cleavage Sites Determination

Cleavage sites determination was performed as described by Pei et al. (2024). Initial PCR was performed using the GeneRacer 5′ outer primer and *ZmCML21* specific 3′ outer primer (5′‐TCAGACCGCGCGCTCCATCAT‐3′). Nested PCR was carried out using the GeneRacer 5′ inner nested primer and *ZmCML21* specific 3′ inner primer (5′‐CTCGAGCCGCCCATCCCGAGGC‐3′). The PCR product was inserted into a pEASY‐T1 vector and sequenced.

### 4.6 Plant Culture and Low Pi Treatment

Maize seeds were sown in low Pi soil (total phosphate concentration is about 0.83 g/kg soil, available phosphate concentration is about 7.13 mg/kg soil) for 3 weeks, and low phosphate tolerance was assessed and watered with non‐Pi nutrient solution every 4 days. For yield character statistics, homogenised low Pi soil (total phosphate concentration is about 0.83 g/kg soil, available phosphate concentration is about 7.13 mg/kg soil) was put in pots, and maize plants were cultivated in low Pi soil until harvest and watered with low phosphate (5 μM KH_2_PO_4_) nutrient solution every 4 days. At harvest time, mature ears were dried and yield traits were determined. Maize plants watered with sufficient phosphate (1 mM KH_2_PO_4_) nutrient solutions acted as a control. The maize plants were grown at 25°C–30°C/20°C–25°C (day/night) with a photoperiod cycle of 14 h of light (500–600 mmol m^−2^ s^−1^) at approximately 65% relative humidity.

### 4.7 Determination of Biomass and P Content of Maize Plants

Root and shoot of maize plants were dried at 80°C to a constant weight for biomass determination. Total P content was determined as described previously (Murphy and Riley 1962; Wei et al. 2019).

### 4.8 Measurement of Root Length, Leaf Length and Anthocyanin Content

The length of crown roots, lateral roots, and leaf was measured using a ruler. Old leaves were extracted with 10 mL of HCl:methanol solution (1:99, v/v) overnight at 4°C. Absorbance was read at 530 and 657 nm; the relative anthocyanin contents were calculated as A530–0.33 A657.

### 4.9 Determination of P Content of Soil

To measure available P contents of soil, 0.5 g of fresh soil sample was weighed and extracted available P by soaking with 0.5 mol · L^−1^ NaHCO_3_ solution. The soil suspension was shaken at 200 rpm for 3 h and filtered. To measure total P contents of soil, 0.5 g soil sample was added to 8 mL of H_2_SO_4_ and 10 drops of HClO_4_, then digested for 1 h. The P contents were determined by the ammonium molybdate ascorbic acid method (Murphy and Riley 1962; Wei et al. 2019).

### 4.10 Measurement of Organic Acid Secretion Rate

Maize seeds were surface sterilised and germinated for 3 days, and then cultured in low phosphate (5 μM KH_2_PO_4_) nutrient solution for 20 days. Plants grown in a normal phosphate (1 mM KH_2_PO_4_) nutrient solution acted as a control. After 20 days of stress, maize roots were cleaned up with distilled water and transferred into thymol solution (5 mg/L) for 5 min. Then maize plants were transferred into 100 mL of CaCl_2_ solution (0.5 mM) for 12 h. Then 20 mL of the root secretion solution was taken and freeze‐dried with a vacuum freeze‐drier. The residue was dissolved in 0.3 mL of 0.1 M HCl solution and filtered with a millipore filter (0.45 μm). The contents of organic acid were analysed using high‐performance liquid chromatography (HPLC) (SHIMADZU5100, Japan) with a UV detector (SHIMADZU, SPD‐10AVP, Japan).

### 4.11 Detection of Rhizosphere Acidification

Maize seeds were surface sterilised and germinated for 3 days, and then cultured in low phosphate (5 μM KH_2_PO_4_) nutrient solution for 20 days. Plants grown in a normal phosphate (1 mM KH_2_PO_4_) nutrient solution acted as a control. After 20 days of stress, the pH value of the nutrient solution was measured using a portable pH metre on the sixth day after the fresh nutrient solution was replaced. The initial pH value of the fresh nutrient solution is 6.0. The visualisation of rhizosphere acidification was conducted as described by Yan et al. (2002). The roots of maize plants stressed for 20 days were carefully spread on an agar medium containing 1% (w/v) agar and 0.06% (w/v) bromocresol purple, and incubated in the dark for 1 h before taking photographs.

### 4.12 Root pH Fluorescence Detection

Root tip pH was detected by a pH imaging technique as described (Zhang et al. 2020). BCECF (2′,7′‐bis‐(2‐carboxyethyl)‐5‐(and‐6)‐carboxyfluorescein) was dissolved in DMSO to make a stock solution and stored at −20°C. Root tips (1 cm) were cut and immersed in BCECF at 5 μM final concentration of 5 μM in buffer solutions (HEPES, pH 7.4) and incubated in the dark for 30 min. Then the root tips were rinsed with deionised water. Samples were excited with 488 nm/458 nm light and the BCECF fluorescence was measured at 530 nm with a confocal laser‐scanning microscope (Zeiss LSM 710, Thuringia, Germany).

### 4.13 Utilisation of Insoluble Phosphate

Maize seeds with uniform size were surface sterilised with 70% (v/v) ethanol and 0.1% (w/v) HgCl_2_, rinsed with sterile water, and germinated at 28°C in darkness for 3 days, then transferred into non‐Pi nutrient solution containing FePO_4_ (1 g/L) for 20 days. Maize plants cultured in normal phosphate (control, 1 mM KH_2_PO_4_) nutrient solutions acted as a control. The nutrient solution was replaced every week. Then, biomass and P content were analysed. The maize plants were grown at 25°C–30°C/20°C–25°C (day/night) with a photoperiod cycle of 14 h of light (500–600 mmol m^−2^ s^−1^) at approximately 65% relative humidity.

### 4.14 Transcriptome Profiles Analysis

Maize seeds (WT and *ZmCML21OE1*) were surface sterilised and germinated for 3 days and then cultured in low phosphate (5 μM KH_2_PO_4_) nutrient solution for 20 days. Then the roots of WT and *ZmCML21OE1* were collected for total RNA extraction. There were three biological replicates for WT and *ZmCML21OE1*, and six RNA libraries were constructed. Sequencing libraries were constructed using NEBNext UltraTM RNA Library Prep Kit for Illumina (NEB, USA). The library preparations were sequenced on an Illumina NovaSeq platform.

### 4.15 Untargeted Metabolome Analyses

Maize seeds (WT and *ZmCML21OE1*) were surface sterilised and germinated for 3 days and then cultured in low phosphate (5 μM KH_2_PO_4_) nutrient solution for 20 days. After 20 days of stress, maize roots were cleaned up with distilled water and transferred into thymol solution (5 mg/L) for 5 min. Then, maize plants were transferred into 100 mL of distilled water for 8 h to collect root secretion. Then 20 mL of the root secretion solution was taken and freeze‐dried with a vacuum freeze‐drier. The residue was dissolved in 0.3 mL of distilled water. The solution was filtered with a 0.22 μm filter and analysed using a Vanquish UHPLC system (Thermo Fisher) coupled with an Orbitrap Q Exactive HF‐X mass spectrometer (Thermo Fisher).

### 4.16 Subcellular Localisation

The *ZmCML21* or *ZmPMA2* coding region was cloned into the pBI221‐GFP vector. Transient expression of GFP‐fused proteins in maize protoplasts was performed as described by Cao et al. (2014). NAA60‐PAN580 vector was used as a PM marker. The fluorescence signal was observed using a confocal laser‐scanning microscope (Leica TCS SP2; Leica Microsystems GmbH).

### 4.17 Yeast Two‐Hybrid (Y2H) Assays

The full length of the coding sequence region of *ZmCML21* was inserted into the pBT3–STE vector as a bait that was then transformed into the yeast strain NMY51. The transformed NMY51 yeast strain was then transformed with the maize membrane system yeast cDNA library and placed on the synthetic dropout (SD) medium–Trp–Leu–Ade. Positive clones were selected for sequencing. The interactions between ZmCML21 and ZmPMA2 were verified by Y2H assay. Auto‐activation was tested by co‐transforming bait and prey constructs with their reciprocal EV.

### 4.18 Luciferase Assay

Full length of *ZmCML21 and* the promoter fragments of *ZmPMA2* were inserted into the 35S:GFP and pGreenII 0800:LUC vectors, respectively. The recombinant vectors were transformed into *N. benthamiana* leaves using the agrobacterial infiltration method ( *A. tumefaciens* strain GV3101). The luciferase signal was detected 72 h post‐transfection using a Tanon 5200 multi‐chemiluminescence imaging system (Tanon Science and Technology Co.).

### 4.19 Co‐Immunoprecipitation (Co‐IP)

*ZmCML21*‐FLAG (pBWA(V)H2STMVΩ‐FLAG‐ZmCML21), *ZmPMA2*‐MYC (pBWA(V)H2STMVΩ‐ZmPMA2‐MYC) and pBWA(V)H2STMVΩ‐CCDB EV were individually transformed into *Ag. tumefaciens* strain GV3101, and the transformed strains were infiltrated into *N. benthamiana* leaves, following the protocol described by Liu et al. (2007). Soluble proteins were extracted on ice in 20 mM HEPES (pH 7.5), 40 mM NaCl, 10 mM MgCl_2_, 0.1% v/v Triton X‐100, 1 mM EDTA, 10% v/v glycerol, 1 × protease inhibitor cocktail and 2 mM PMSF, after which the lysate was centrifuged (10,000 *g*, 4°C, 20 min). The resulting supernatant was incubated at 4°C overnight in 40 μL anti‐MYC M2 affinity gel (Sigma, Germany) or anti‐FLAG antibody, and the resulting antigen–antibody complex was collected by centrifugation (6000 *g*, 4°C, 2 min). The beads were washed three times in the extraction buffer, then boiled in SDS buffer. Samples were separated via SDS‐PAGE and probed with either anti‐FLAG or anti‐MYC antibody (Kangwei, Beijing, China).

### 4.20 Measurement of Plasma Membrane (PM) H^+^ ‐ATPase Activity

PM vesicles were isolated from fresh maize roots using the Minute Plant PM protein isolation kit (INVENT, Plymouth MA, USA) and quantified using the BCA protein determination method (Yang et al. 2010). The reaction was performed in 2 mL of solution containing 50 μg/mL PM protein, 10 μM quinacrine, 3 mM MgSO_4_, 100 mM KCl, 250 mM mannitol, 3 mM ATP, and 25 mM BTP‐MES‐HEPES (pH 6.5). The mixed solution was placed in the dark at 26°C and allowed to react for 5 min; the fluorescence was obtained with a fluorescence spectrophotometer (Hitachi, Tokyo, Japan) at 430 nm excitation and 500 nm emission wavelengths. 10 μM carbonyl cyanide 3‐chlorophenylhydrazone (CCCP) was added to eliminate the remaining pH gradient. The PM H^+^‐ATPase activity was measured by the change of fluorescence per unit PM protein per unit reaction time (ΔF/min/mg PM protein).

### 4.21 Statistical Analysis

Statistical analysis was performed using SPSS Statistics 23 software. Data were expressed as means ± SD (standard deviation). All experiments were repeated three times, and all data were analysed using Duncan's multiple‐range test, and statistical significances were considered at the *p* < 0.05 level.

## Author Contributions

H.L. and L.P. planned and designed the research; L.P. and Y.Y. performed the research; L.P., Z.W., and N.L. analysed data; L.P. wrote the paper; Z.D. revised the paper. All authors read and approved the final manuscript.

## Conflicts of Interest

The authors declare no conflicts of interest.

## Supporting information


**Figure S1:** Generation and molecular analysis of ZmmiR1432 transgenic maize plants. (a) Vector for ZmmiR1432 overexpression lines and ZmmiR1432 antisense lines. (b) Polymerase chain reaction (PCR) analysis of wild‐type and transgenic lines with specific primers for bar Lane M, DNA marker DL2000. Lane WT, wild type. (c) Relative expression levels of ZmmiR1432 in transgenic maize plants. Maize roots from ZmmiR1432 transgenic plants were collected to analyse ZmmiR1432 expression levels by real‐time PCR. The expression levels of ZmmiR1432 were normalised to that of maize 5S rRNA. Values are means ± SD of three biological replicates.

**Figure S2:** Leaf anthocyanin content. Maize seeds were sown in low Pi soil, and maize plants watered with sufficient phosphate (1 mM KH_2_PO_4_) nutrient solutions acted as controls. After 3 weeks of growth, old leaves were collected to measure anthocyanin content. Different lowercase letters indicate the statistically significant difference in the same tissue between maize plants under the same Pi conditions at the *p* < 0.05 level using Duncan's multiple‐range test. Values are means ± SD of three biological replicates.

**Figure S3:** The expression pattern of potential target genes of ZmmiR1432. Seven‐day‐old WT maize seedlings were transferred to low phosphate (5 μM KH_2_PO_4_) nutrient solution. After different stress times (0, 1, 5, 10, 15 days), root tips of maize plants were collected to analyse expression levels of *ZmCML21* (a) and calcium‐transporting ATPase coding gene (b) by real‐time PCR. Seven‐day‐old maize seedlings, including WT, anti‐miR1432 lines and OXmiR1432 maize lines, were transferred to low phosphate (5 μM KH_2_PO_4_) nutrient solution for 2 weeks, then root tips were collected and relative expression levels of *ZmCML21* (c) and calcium‐transporting ATPase coding gene (d) were measured. The expression levels were normalised to that of maize *Actin1*. Different lowercase letters indicate the statistically significant difference in the same tissue between maize plants under the same Pi conditions at the *p* < 0.05 level using Duncan's multiple‐range test. Values are means ± SD of three biological replicates.

**Figure S4:** Generation and molecular analysis of *ZmCML21* transgenic maize plants. (a) Vector for *ZmCML21* overexpression lines and *ZmCML21* RNAi lines. (b) Polymerase chain reaction (PCR) analysis of wild type and transgenic lines with specific primers for *ZmCML21* and *bar*. Lane M, DNA marker DL2000. Lane WT, wild type. (c) Relative expression levels of *ZmCML21*. Maize roots from *ZmCML21* transgenic plants were collected to analyse expression levels of *ZmCML21* by real‐time PCR. The expression levels of *ZmCML21* were normalised to that of maize *Actin1*. Values are means ± SD of three biological replicates.

**Figure S5:** Available Pi content of rhizosphere soil. Maize seeds of offspring were sown in low Pi soil (total phosphate concentration is about 0.83 g/kg soil, available phosphate concentration is about 7.13 mg/kg soil) and watered with low phosphate (low Pi, 5 μM KH_2_PO_4_) nutrient solution every 3 days, maize plants watered with sufficient phosphate (1 mM KH_2_PO_4_) nutrient solutions acted as controls. At harvest time, available Pi content of rhizosphere soil was determined. Different lowercase letters indicate the statistically significant difference between maize plants under the same Pi conditions at the *p* < 0.05 level using Duncan's multiple‐range test. Values are means ± SD of three biological replicates.

**Figure S6:** Relative expression levels of genes involved in organic acids synthesis and metabolism. 7‐day‐old maize seedlings, including WT, *ZmCML21OE* lines and *ZmCML21*RNAi maize lines, were transferred to low phosphate (5 μM KH_2_PO_4_) nutrient solution for 3 weeks, then root tips were collected to analyse relative expression levels of genes involved in organic acids synthesis and metabolism by real‐time PCR. The expression levels were normalised to that of maize *Actin1*. Different lowercase letters indicate the statistically significant difference between maize plants under the same Pi conditions at the *p* < 0.05 level using Duncan's multiple‐range test. Values are means ± SD of three biological replicates.

**Figure S7:** Generation and molecular analysis of *ZmPMA2* transgenic maize plants. (a) Vector for *ZmPMA2* overexpression lines. (b) Polymerase chain reaction (PCR) analysis of wild type and transgenic lines with specific primers for *ZmPMA2* and *bar*. Lane M, DNA marker DL2000. Lane WT, wild type. (c) Relative expression levels of *ZmPMA2*. Maize roots from WT and *ZmPMA2* transgenic plants were collected to analyse expression levels by real‐time PCR. The expression levels of *ZmPMA2* were normalised to that of maize *Actin1*. (d) PM H^+^‐ATPase activity in roots of WT and *ZmPMA2* transgenic plants. Lowercase letters indicate the statistically significant difference between maize plants under same Pi conditions at the *p* < 0.05 level using Duncan's multiple‐range test. Values are means ± SD of three biological replicates.

**Table S1:** Preliminary identification of the ZmmiR1432 target genes.

**Table S2:** Prediction of potential complementary miRNAs targeting the *ZmCML21*.

**Table S3:** Transcriptome profiles analysis between *ZmCML21OE1* and WT plants.

**Table S4:** Gene Ontology (GO) enrichment analysis.

**Table S5:** KEGG analysis of differentially expressed genes between *ZmCML21OE1* and WT.

**Table S6:** Metabolomics analysis between *ZmCML21OE1* and WT plants.

**Table S7:** KEGG analysis of differential metabolites between *ZmCML21OE1* and WT plants.

**Table S8:** Two‐hybrid (Y2H) screen of ZmCML21.

**Table S9:** PCR primers used in this study.

## Data Availability

RNA‐Seq data have been deposited in NCBI's Sequence Read Archive database (https://www.ncbi.nlm.nih.gov/sra/) under accession number PRJNA1200251.
