# Supplementary figures and images for: ZmmiR1432‐ ZmCML21 ‐ ZmPMA2 Module Affects Maize Low Phosphate Tolerance via Regulating Organic Acid Secretion
Source: Plant Biotechnol J. 2025 Oct 6;24(2):921–38. doi: 10.1111/pbi.70385 (PMC12906843; doi:10.1111/pbi.70385)

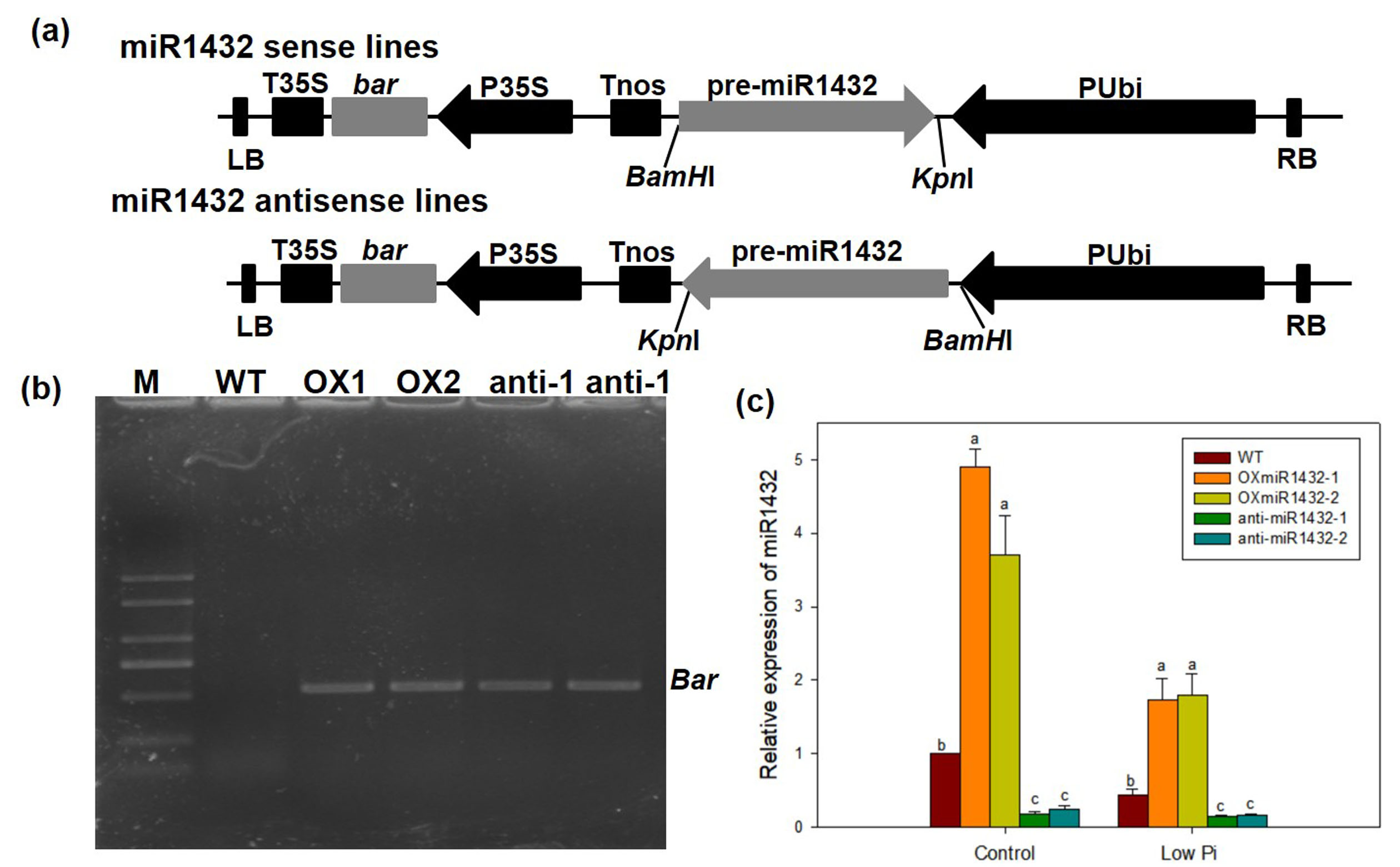

Supplement: Supplementary file 1 — Figure S1: Generation and molecular analysis of ZmmiR1432 transgenic maize plants. (a) Vector for ZmmiR1432 overexpression lines and ZmmiR1432 antisense lines. (b) Polymerase chain reaction (PCR) analysis of wild‐type and transgenic lines with specific primers for bar Lane M, DNA marker DL2000. Lane WT, wild type. (c) Relative expression levels of ZmmiR1432 in transgenic maize plants. Maize roots from ZmmiR1432 transgenic plants were collected to analyse ZmmiR1432 expression levels by real‐time PCR. The expression levels of ZmmiR1432 were normalised to that of maize 5S rRNA. Values are means ± SD of three biological replicates. [file PBI-24-921-s009.jpg]

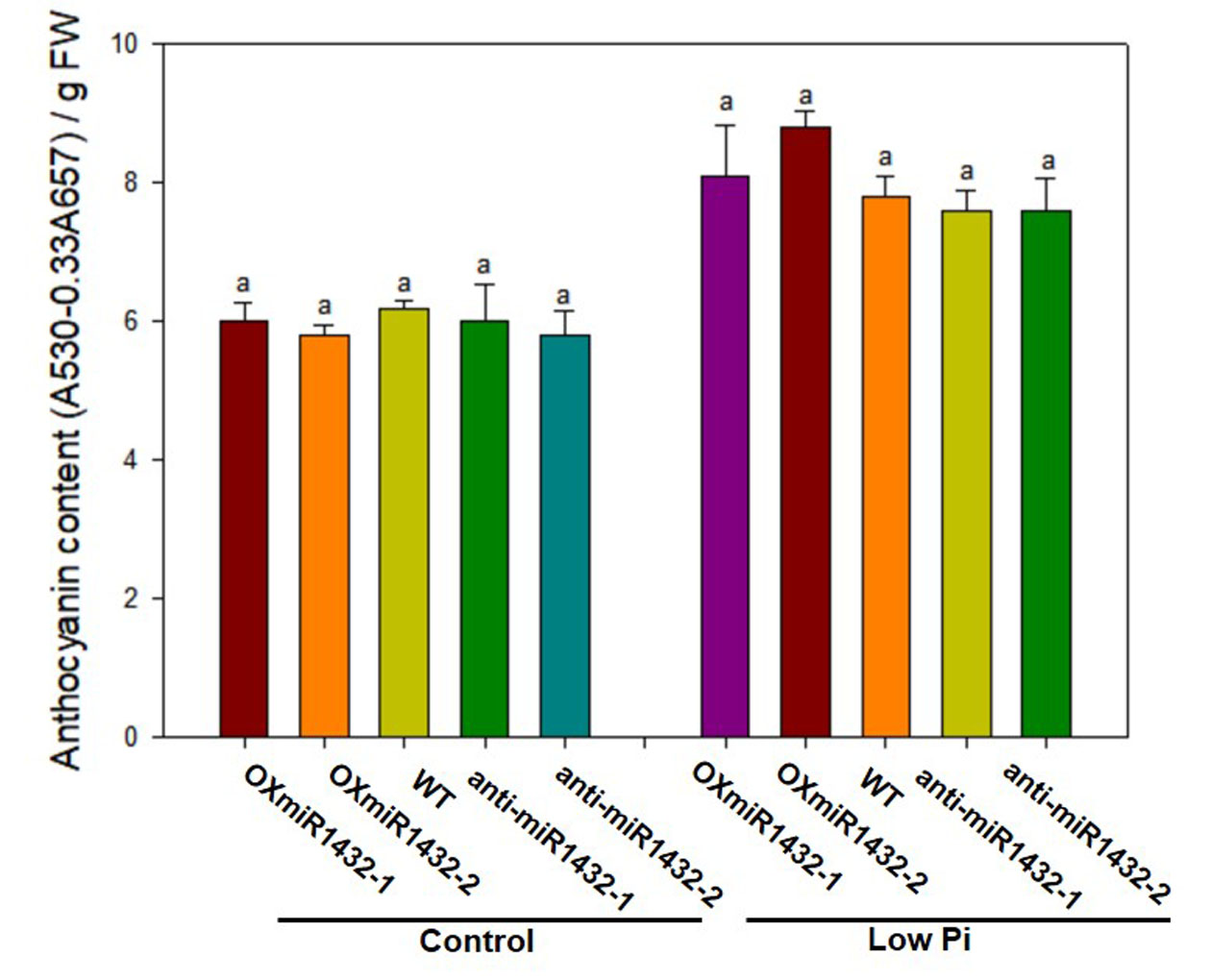

Supplement: Supplementary file 2 — Figure S2: Leaf anthocyanin content. Maize seeds were sown in low Pi soil, and maize plants watered with sufficient phosphate (1 mM KH2PO4) nutrient solutions acted as controls. After 3 weeks of growth, old leaves were collected to measure anthocyanin content. Different lowercase letters indicate the statistically significant difference in the same tissue between maize plants under the same Pi conditions at the p < 0.05 level using Duncan's multiple‐range test. Values are means ± SD of three biological replicates. [file PBI-24-921-s005.jpg]

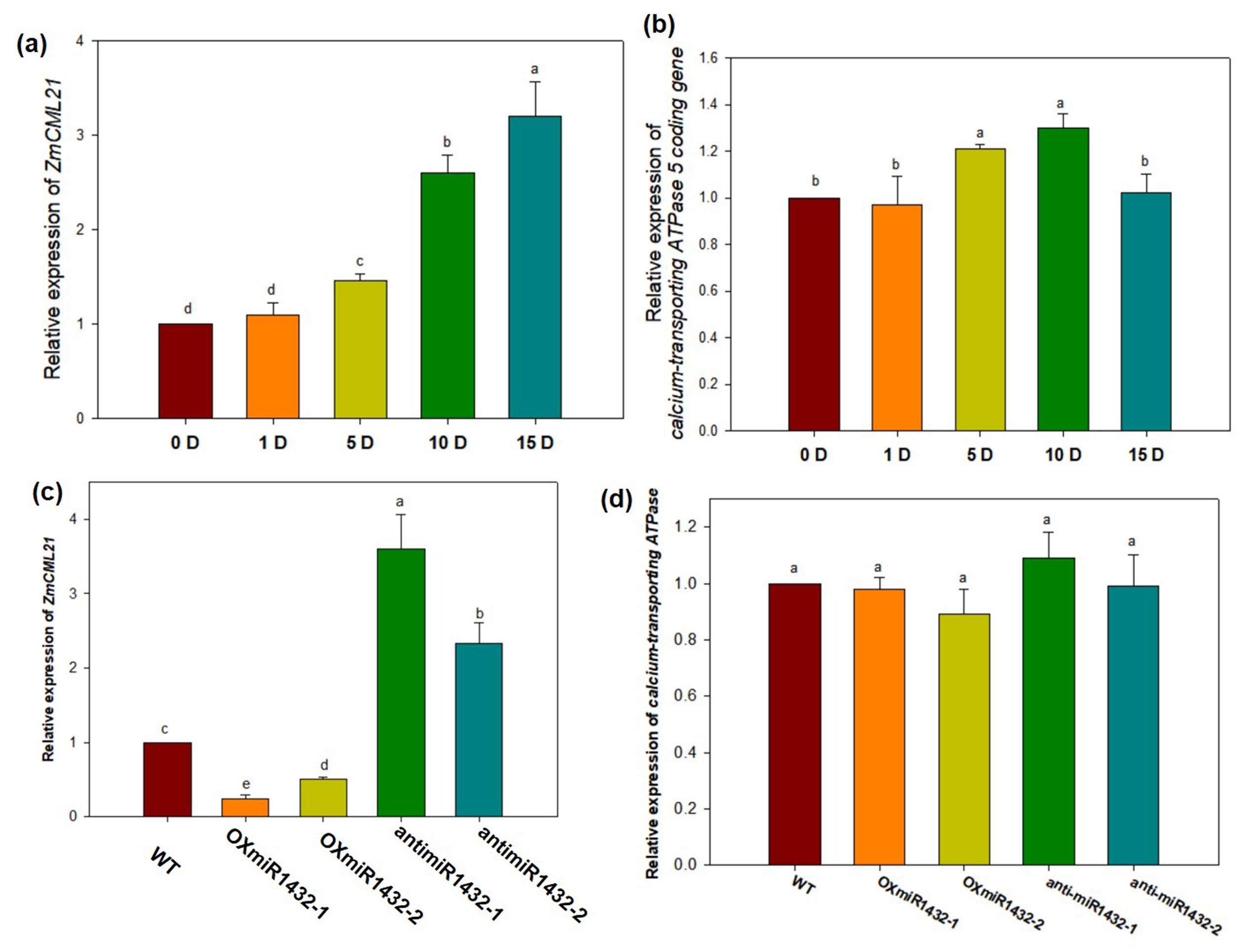

Supplement: Supplementary file 3 — Figure S3: The expression pattern of potential target genes of ZmmiR1432. Seven‐day‐old WT maize seedlings were transferred to low phosphate (5 μM KH2PO4) nutrient solution. After different stress times (0, 1, 5, 10, 15 days), root tips of maize plants were collected to analyse expression levels of ZmCML21 (a) and calcium‐transporting ATPase coding gene (b) by real‐time PCR. Seven‐day‐old maize seedlings, including WT, anti‐miR1432 lines and OXmiR1432 maize lines, were transferred to low phosphate (5 μM KH2PO4) nutrient solution for 2 weeks, then root tips were collected and relative expression levels of ZmCML21 (c) and calcium‐transporting ATPase coding gene (d) were measured. The expression levels were normalised to that of maize Actin1. Different lowercase letters indicate the statistically significant difference in the same tissue between maize plants under the same Pi conditions at the p < 0.05 level using Duncan's multiple‐range test. Values are means ± SD of three biological replicates. [file PBI-24-921-s015.jpg]

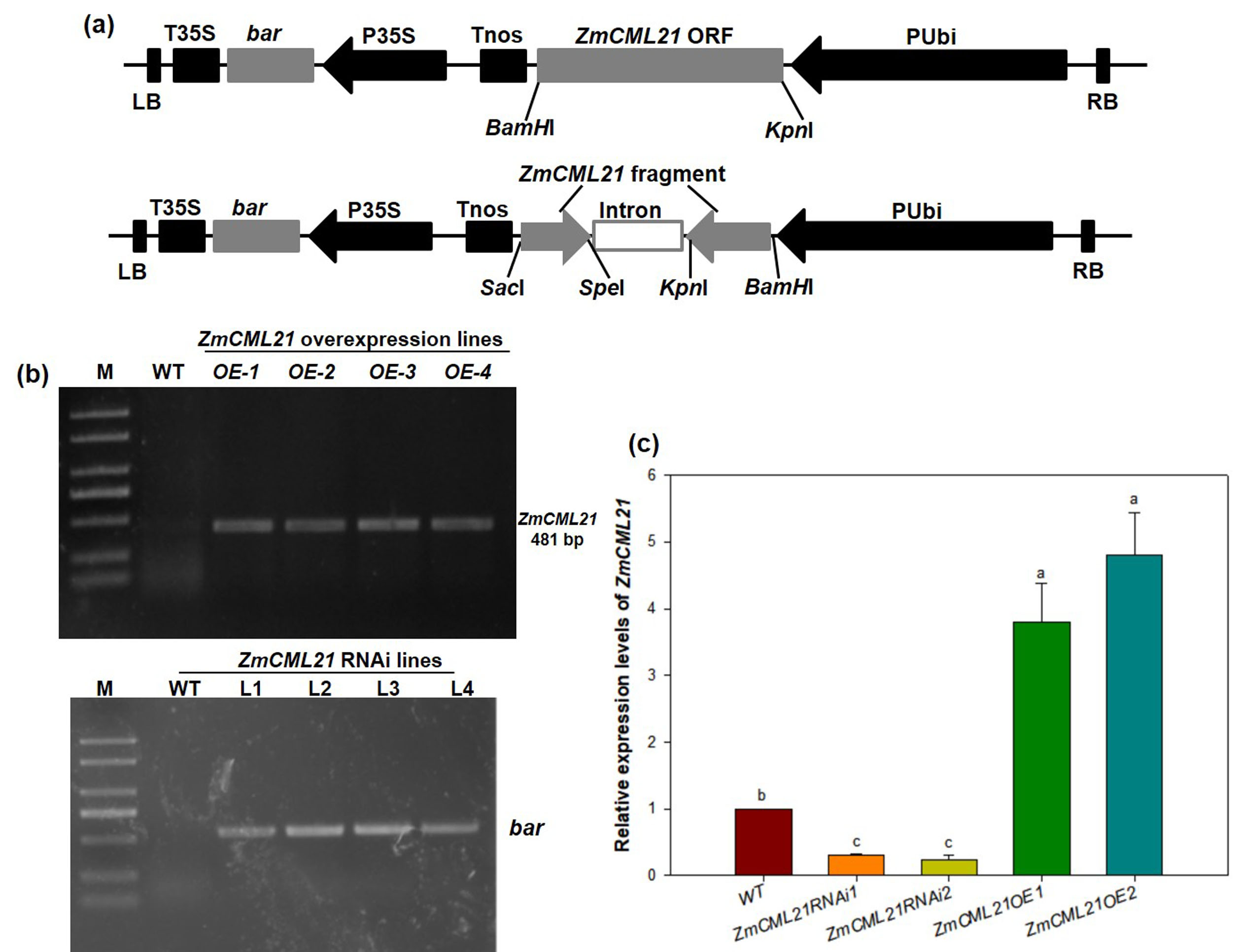

Supplement: Supplementary file 4 — Figure S4: Generation and molecular analysis of ZmCML21 transgenic maize plants. (a) Vector for ZmCML21 overexpression lines and ZmCML21 RNAi lines. (b) Polymerase chain reaction (PCR) analysis of wild type and transgenic lines with specific primers for ZmCML21 and bar. Lane M, DNA marker DL2000. Lane WT, wild type. (c) Relative expression levels of ZmCML21. Maize roots from ZmCML21 transgenic plants were collected to analyse expression levels of ZmCML21 by real‐time PCR. The expression levels of ZmCML21 were normalised to that of maize Actin1. Values are means ± SD of three biological replicates. [file PBI-24-921-s013.jpg]

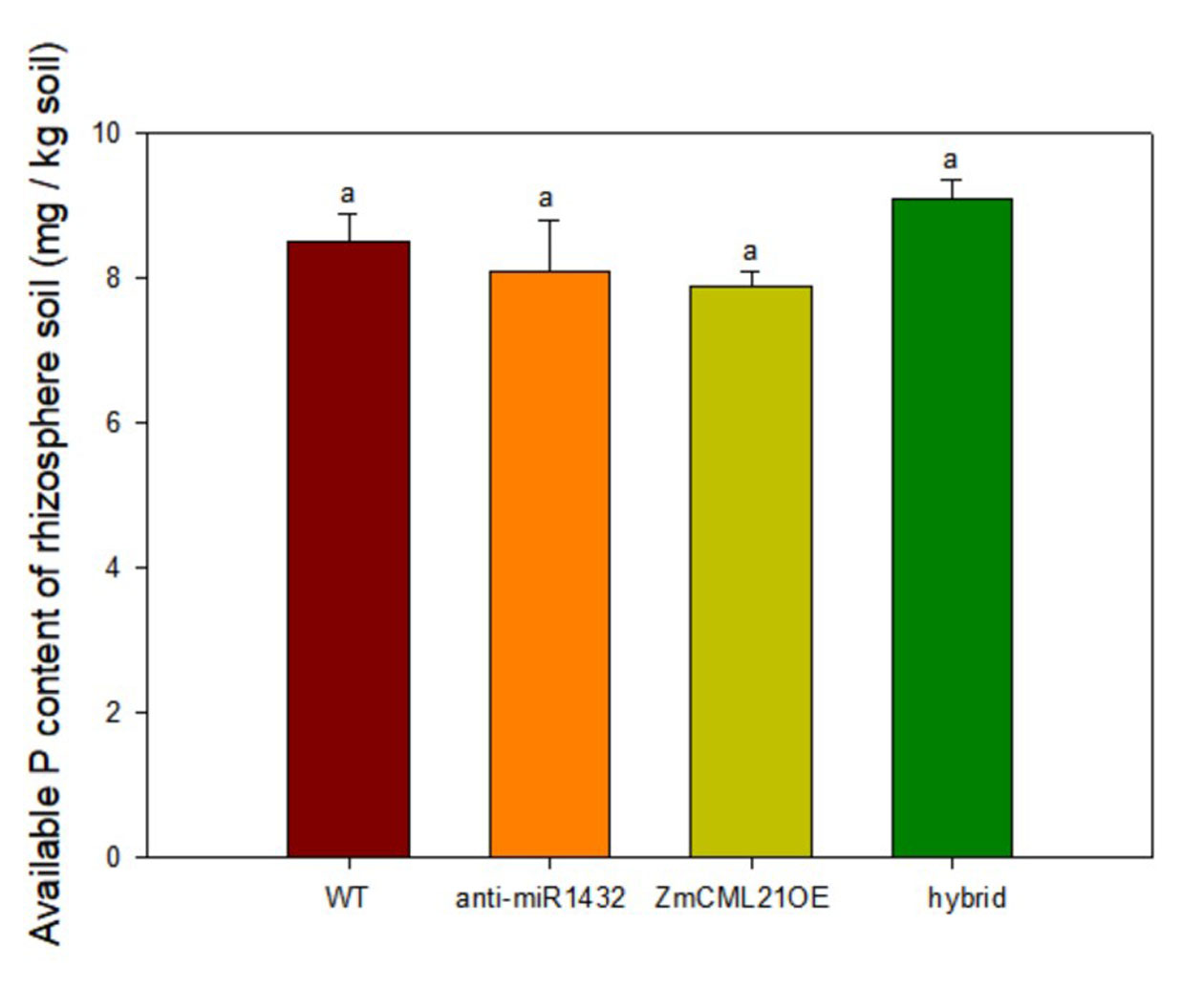

Supplement: Supplementary file 5 — Figure S5: Available Pi content of rhizosphere soil. Maize seeds of offspring were sown in low Pi soil (total phosphate concentration is about 0.83 g/kg soil, available phosphate concentration is about 7.13 mg/kg soil) and watered with low phosphate (low Pi, 5 μM KH2PO4) nutrient solution every 3 days, maize plants watered with sufficient phosphate (1 mM KH2PO4) nutrient solutions acted as controls. At harvest time, available Pi content of rhizosphere soil was determined. Different lowercase letters indicate the statistically significant difference between maize plants under the same Pi conditions at the p < 0.05 level using Duncan's multiple‐range test. Values are means ± SD of three biological replicates. [file PBI-24-921-s002.jpg]

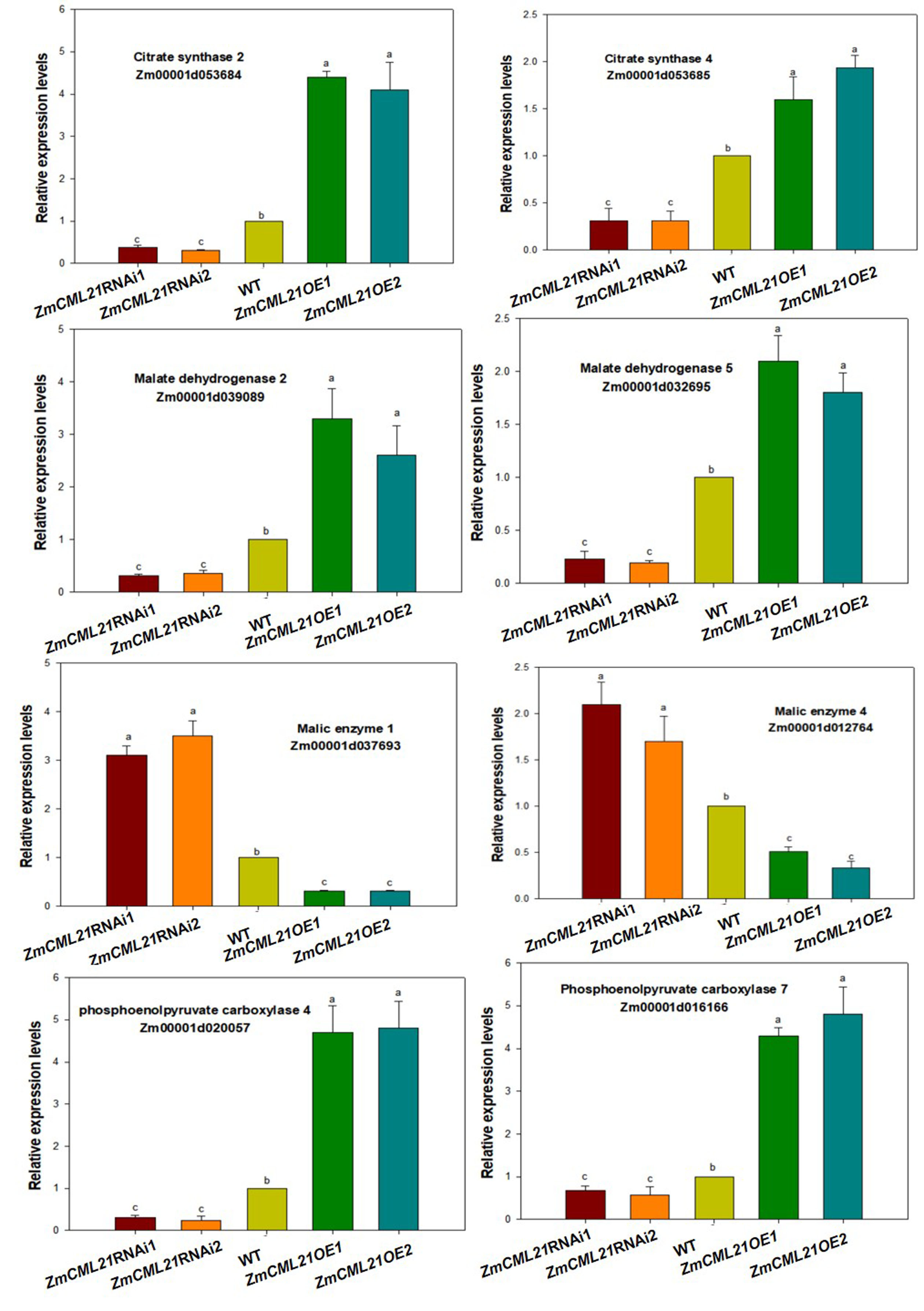

Supplement: Supplementary file 6 — Figure S6: Relative expression levels of genes involved in organic acids synthesis and metabolism. 7‐day‐old maize seedlings, including WT, ZmCML21OE lines and ZmCML21RNAi maize lines, were transferred to low phosphate (5 μM KH2PO4) nutrient solution for 3 weeks, then root tips were collected to analyse relative expression levels of genes involved in organic acids synthesis and metabolism by real‐time PCR. The expression levels were normalised to that of maize Actin1. Different lowercase letters indicate the statistically significant difference between maize plants under the same Pi conditions at the p < 0.05 level using Duncan's multiple‐range test. Values are means ± SD of three biological replicates. [file PBI-24-921-s001.jpg]

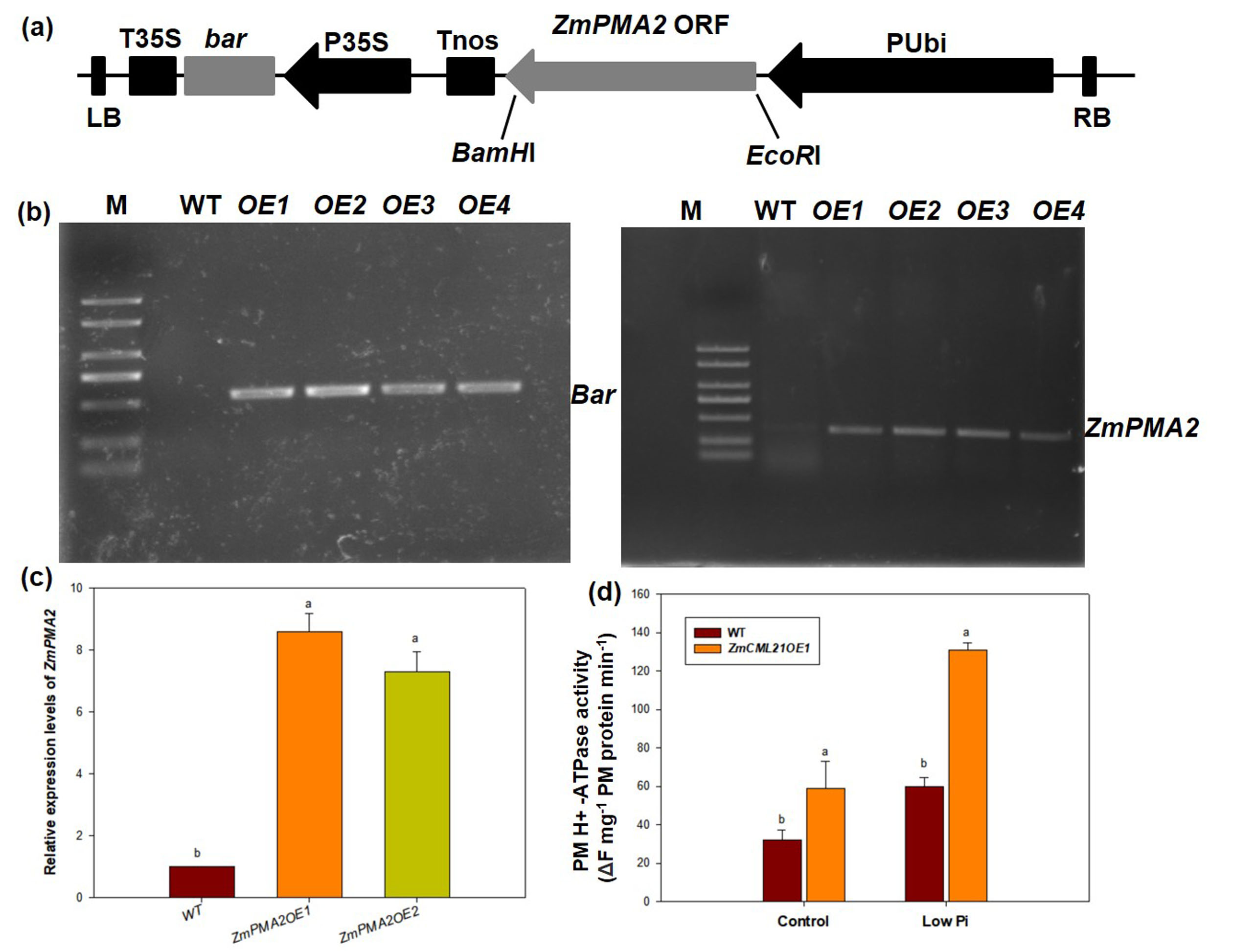

Supplement: Supplementary file 7 — Figure S7: Generation and molecular analysis of ZmPMA2 transgenic maize plants. (a) Vector for ZmPMA2 overexpression lines. (b) Polymerase chain reaction (PCR) analysis of wild type and transgenic lines with specific primers for ZmPMA2 and bar. Lane M, DNA marker DL2000. Lane WT, wild type. (c) Relative expression levels of ZmPMA2. Maize roots from WT and ZmPMA2 transgenic plants were collected to analyse expression levels by real‐time PCR. The expression levels of ZmPMA2 were normalised to that of maize Actin1. (d) PM H+‐ATPase activity in roots of WT and ZmPMA2 transgenic plants. Lowercase letters indicate the statistically significant difference between maize plants under same Pi conditions at the p < 0.05 level using Duncan's multiple‐range test. Values are means ± SD of three biological replicates. [file PBI-24-921-s011.jpg]

**Table S1.** Preliminary identification of the ZmmiR1432 target genes.


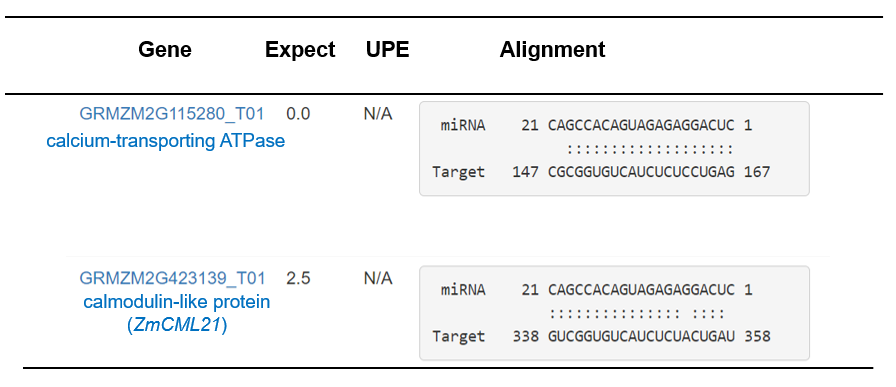

Supplement: Supplementary file 8 — Table S1: Preliminary identification of the ZmmiR1432 target genes. [file PBI-24-921-s006.docx]

**Table S2.** Prediction of potential complementary miRNAs targeting the *ZmCML21.*


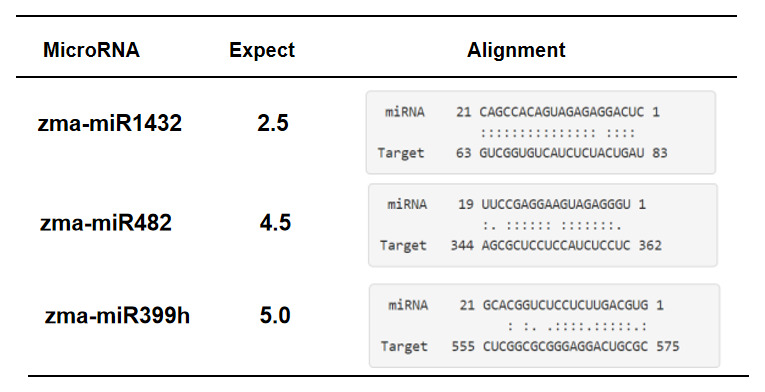

Supplement: Supplementary file 9 — Table S2: Prediction of potential complementary miRNAs targeting the ZmCML21. [file PBI-24-921-s007.docx]
