# Supplementary material for: ZmmiR1432‐ ZmCML21 ‐ ZmPMA2 Module Affects Maize Low Phosphate Tolerance via Regulating Organic Acid Secretion
Source: Plant Biotechnol J. 2025 Oct 6;24(2):921–38. doi: 10.1111/pbi.70385 (PMC12906843; doi:10.1111/pbi.70385)
Supplement: Supplementary file 16 — Table S9: PCR primers used in this study. [file PBI-24-921-s014.docx]

**Table S9.** PCR primers used in this study.

| **Primer** | **Sequence 5’-3’** | **Description** |
| --- | --- | --- |
| miR1432 sense-F  miR1432 sense-R | CGGGGTACCGCTCAGGAGAGATGACACCG  CGCGGATCCTGCTTCAGGCGAGATGACAC | Used for miR1432 sense vector construction |
| miR1432 antisense-F  miR1432 antisense-R | CGCGGATCCGCTCAGGAGAGATGACACCG  CGGGGTACCTGCTTCAGGCGAGATGACAC | Used for miR1432 antisense vector construction |
| *bar* PCR-F  *bar* PCR-R | ATGAGCCCAGAACGACGCC  TCAAATCTCGGTGACGGGC | Used for miR1432 transgenic lines identification |
| miR1432 forward primers | CTCAGGAGAGATGACACCGAC | Used for real-time RT-PCR |
| 5S rRNA forward primers | GATCCCATTCCGACCTCGATATA | Used for real-time RT-PCR |
| pri-miR1432 qRT PCR-F  pri-miR1432 qRT PCR-R | AGATGACACCGACGCCAGAC  GCGAGATGACACCCACTTCA | Used for real-time RT-PCR |
| *Actin* qRT PCR-F  *Actin* qRT PCR-R | ATCACCATTGGGTCAGAAAGG  GTGCTGAGAGAAGCCAAAATAGA | Used for real-time RT-PCR |
| *ZmCML21* F  *ZmCML21* R | CCGGCGCTGGACCTCCTCCT  GCTCGCAATGCCGCGGGGGA | Used for *N. benthamiana* transient expression |
| *ZmCML21* cloning-F  *ZmCML21* cloning-R | ATGATCCCCTCGCTCGAT  AGGCTCCCGTTTTCTTGG | Used for *ZmCML21* clone |
| *ZmCML21* RNAi-F1  *ZmCML21* RNAi-R1 | CGCGGATCCATGTTGGAGCTGCTGGTGCT  CGGGGTACCCACGGCCGCCACGGTGCCCG | Used for *ZmCML21* RNAi vector construction |
| *ZmCML21* RNAi-F2  *ZmCML21* RNAi-R2 | GAGTACTATGTTGGAGCTGCTGGTGCT  CGGACTAGTCACGGCCGCCACGGTGCCCG | Used for *ZmCML21* RNAi vector construction |
| *ZmCML21 OE*-F  *ZmCML21 OE*-R | CGGGGTACCATGTTGGAGCTGCTGGTGCTG  CGCGGATCCTCAGACCGCGCGCTCCATCATG | Used for *ZmCML21* overexpression vector construction |
| *ZmCML21* qRT PCR-F  *ZmCML21* qRT PCR-R | TCATCGTGTCGGTGTCATCT  TCCCTGAACTCGCGGAAGCT | Used for real-time RT-PCR |
| *ZmCML21* PCR-F1  *ZmCML21* PCR-R1 | TCATCGTGTCGGTGTCATCT  AGGCGGCGCAGCACGTACCAG | Used for *ZmCML21* overexpression maize lines identification |
| *ZmCML21* 3’ outer primer  *ZmCML21* 3’ inner primer | TCAGACCGCGCGCTCCATCAT  CTCGAGCCGCCCATCCCGAGGC | Used for 5’ RNA ligase-mediated RACE to detect cleavage sites |
| *ZmPMA2* cloning-F  *ZmPMA2* cloning-R | TAGGGCAAAGTGAAGAAG  GAGTTGAGGGTAGTATGGT | Used for *ZmPMA2* clone |
| *ZmPMA2OE*-F  *ZmPMA2OE*-R | GAATTCATGAGTTTATTAGGCCTTTTG  GGATCCCTAGATGTCCAGACCCTTCTGC | Used for *ZmPMA2* overexpression vector construction |
| *ZmPMA2* PCR-F1  *ZmPMA2* PCR-R1 | CCGCCCAGGATGACTTTC  CTCCCTTTCTTCCGTGCC | Used for *ZmPMA2* overexpression maize lines identification |
